# Transcriptomic Analysis of *Aedes aegypti* Innate Immune System in Response to Ingestion of Chikungunya Virus

**DOI:** 10.3390/ijms20133133

**Published:** 2019-06-27

**Authors:** Liming Zhao, Barry W. Alto, Yongxing Jiang, Fahong Yu, Yanping Zhang

**Affiliations:** 1 Florida Medical Entomology Laboratory, University of Florida, 200 9th Street South East, Vero Beach, FL 32962, USA; 2 Mosquito Control Services, City of Gainesville, 405 NW 39th Avenue Gainesville, FL 32609, USA; 3 Interdisciplinary Center for Biotechnology Research, University of Florida, 2033 Mowry Road, Gainesville, FL 32611, USA

**Keywords:** *Aedes aegypti*, transcriptome, immune responses, chikungunya virus, anti-microbial peptide, gene expression

## Abstract

*Aedes aegypti* (L.) is the primary vector of emergent mosquito-borne viruses, including chikungunya, dengue, yellow fever, and Zika viruses. To understand how these viruses interact with their mosquito vectors, an analysis of the innate immune system response was conducted. The innate immune system is a conserved evolutionary defense strategy and is the dominant immune system response found in invertebrates and vertebrates, as well as plants. RNA-sequencing analysis was performed to compare target transcriptomes of two Florida *Ae. aegypti* strains in response to chikungunya virus infection. We analyzed a strain collected from a field population in Key West, Florida, and a laboratory strain originating from Orlando. A total of 1835 transcripts were significantly expressed at different levels between the two Florida strains of *Ae. aegypti*. Gene Ontology analysis placed these genes into 12 categories of biological processes, including 856 transcripts (up/down regulated) with more than 1.8-fold (*p*-adj (*p*-adjust value) ≤ 0.01). Transcriptomic analysis and q-PCR data indicated that the members of the *AaeCECH* genes are important for chikungunya infection response in *Ae. aegypti*. These immune-related enzymes that the chikungunya virus infection induces may inform molecular-based strategies for interruption of arbovirus transmission by mosquitoes.

## 1. Introduction

*Aedes aegypti* (L.) is a major vector of arboviruses including chikungunya, dengue, yellow fever, and Zika. Chikungunya virus (CHIKV) is an emerging viral disease in the family Togaviridae, genus *Alphavirus*, which is transmitted to humans by infected mosquitoes, primarily *Ae. aegypti* and *Ae. albopictus*. Recent outbreaks of CHIKV occurred in Kenya in 2004 (East/Central/South African, ECSA, CHIKV lineage) and the island of La Réunion in 2005–2006 (Indian Ocean CHIKV lineage). CHIKV outbreaks were also documented in Gabon on the west coast of Central Africa (East Central South Africa lineage) in 2007 and in 2010 [1]. Later, CHIKV emerged in the New World in 2013 on St. Martin Island (Asian CHIKV lineage) and continued to spread throughout the Americas [2,3,4]. Other *Aedes* species that have been reported to transmit CHIKV include *Ae. vittatus* and *Ae. koreicus*. *Aedes vittatus* has a geographical distribution throughout tropical Asia, Africa, and the Mediterranean region of Europe [5], while *Ae. koreicus* is new to Europe with a geographical distribution consisting of tropical and sub-tropical climates across the globe [6]. More than four million cases of human infection involving CHIKV have occurred worldwide over the past 12 years, making mosquito control and interruption of CHIKV transmission a priority [7]. Common symptoms associated with human infection include: fever, headache, muscle pain, rash, and induced joint damage [8,9], with the possibility of chronic musculoskeletal diseases [10] and chronic arthritis [11]. There is no vaccine currently available for the prevention of CHIKV and so controlling the mosquito vectors is considered the primary method for reducing the risk of transmission.

The innate immunity is an evolutionarily conserved defense system in invertebrates, vertebrates, and plants. It is a host response that serves as the first line of defense via quorum sensing, or by sensing pathogen-associated molecular patterns through germline-encoded pattern recognition receptors [12]. Aside from the innate immune system, vertebrates possess an adaptive (acquired) immune system which arose 500 million years ago in ectothermic (cold-blooded) vertebrates [13]. Mosquitoes lack an adaptive immune system [14] and solely rely on mounting an innate immune response to defend against infection, including pathogens and parasites encountered through the ingestion of blood [15,16,17,18,19,20]. Mosquito and mosquito cell lines produce humoral and cellular components as part of their innate immune responses against invading pathogens and parasites [21,22,23,24,25].

Mosquitoes respond to infection using an array of molecular signaling pathways and immune effector proteins. Transcriptomic profiling of the immune system response in *Ae. aegypti* has revealed genome-wide mechanisms that are implicated to defend against arbovirus infections [17,26,27,28,29]. Gene expression profiling in response to infections of arboviruses, including chikungunya, dengue, West Nile and Zika viruses, have been performed in *Ae. aegypti, Culex pipiens quinquefasciatus,* and other mosquito species [17,27,28,29,30,31,32,33]. Many genes are involved in the mosquito’s antiviral immunity, including antimicrobial peptide genes and defensins [34,35,36,37]. Immune responses and some arthropod immunity pathways such as Toll, Imd, JAK/STAT, Leucine-rich repeat (LRR) proteins, and RNAi play central roles during mosquito arboviral infection [17,28,29,31,38,39,40,41,42,43]. An infection study suggested that genes encoding trypsins, metalloproteinases, and serine-type endopeptidases may be involved in midgut escape barriers in *Ae. aegypti* infected with CHIKV [17]. Another study revealed that the thioester-containing proteins (TEP) are positive regulators of the functional integration between the immune and circulatory systems of mosquitoes and can reduce pathogen infection intensity [44]. A study on dengue virus infection in *Ae. aegypti* characterized changes in expression of a member of Pacifastin family (serine protease inhibitors) involved in immune responses, including prophenoloxidase cascade, antibacterial, and antifungal defenses [45]. Arbovirus infection may also be associated with changes in the expression of other categories of biological processes, such as arbovirus infection changes in *Ae. aegypti* blood feeding behavior and oviposition olfactory preferences [46,47,48].

Transcriptomic studies have been conducted to elucidate the altered functional pathways in response to viral infection between populations/strains of mosquitoes [29,30]. The transcriptome of *Ae. aegypti* suggested that most detoxification enzymes and immune system enzymes showed different gene expression patterns between two strains of *Ae. aegypti* in response to Zika virus infection [29]. Three genetically polymorphic and geographically distinct *Ae. aegypti* populations showed differences in gene expression profiles for transcripts that encode proteins associated with catalytic activities, molecular transport, metabolism of lipids, and functions related to blood digestion in blood-fed mosquitoes [49].

The present study aims to improve our understanding of the entomological components of CHIKV epidemiology in the context of molecular responses of a mosquito vector in response to infection through a combination of traditional genetic and biochemical approaches. Transcriptomic studies have the potential to provide insight into novel molecular strategies that may be used to improve public health through the interruption of arbovirus transmission by mosquito vectors.

## 2. Results

### 2.1. Global Changes in Transcriptome of the Aedes aegypti Female Adult in Response to CHIKV Infection

*Ae. aegypti* is genetically polymorphic, as shown by differences among distinct geographic populations (strains). To understand the molecular interactions of the arbovirus with a natural population of *Ae. aegypti* from Florida, RNA-sequencing was conducted to explore the global changes in the *Ae. aegypti* (Key West and Orlando populations/strains) transcriptome in response to oral ingestion of CHIKV-infected blood and subsequent infection. In this study, four-day-old adult females of *Ae. aegypti* were fed a blood meal containing 8.0 ± 0.09 and 8.3 ± 0.08 log10 plaque forming units (pfu)/mL of CHIKV (Table 1). Fresh-fed (3 h post infection) mosquitoes ingested 4.89 to 5.68 log10 pfu/mL of CHIKV. The highest mosquito body titers observed for both strains occurred 5 days post infection (Key West, 5.87 ± 0.39 and Orlando, 4.78 ± 0.39 log10 pfu/mL, Table 1). A two-way ANOVA showed a significant strain (F_1,59_ = 26.04, *p* < 0.0001), time (F_5,59_ = 17.40, *p* < 0.0001), and strain by time interaction (F_5,59_ = 5.85, *p* = 0.0002). Pairwise comparisons between time points for the Key West strain showed significantly higher mosquito body viral titers at 5 days and 7 days post infection compared to 1 day post infection (Table 1). The remaining comparisons of the time points for the Key West strain were not significantly different from one another. Pairwise comparisons between time points for the Orlando strain had significantly higher mosquito body viral titers at 5 days post infection compared to 1 day post infection. Viral titers at 2 days post infection were lower than all other time points (Table 1). Comparisons between strains at each time point (e.g., Key West versus Orlando at 1 day post infection) showed similar titers for all time points except for 2 days post infection, where the Orlando strain had significantly lower viral titer than the Key West strain (Table 1).

### 2.2. Expression Profiles of DE (Differentially Expressed) Transcripts in Response to Chikungunya Virus (CHIKV) Infection in Aedes aegypti Key West Population/Strain

RNA was extracted from female *Ae. aegypti* for the time course study, first at three hours post ingestion and again at three days post ingestion (dpi). A total of 18 RNA-seq libraries were created using female *Ae. aegypti* infected by CHIKV (3 h and 3 dpi) and the control (fed uninfected blood, 3 h and 3 dpi), using three replicates of each group. A total of 706,051,842 raw reads were generated from the Orlando strain. The cleanup resulted in 187,384,965 cleaned reads, which mapped to 18,840 transcripts of *Ae. aegypti* (Table S1). A total of 797,720,788 raw reads were generated from the Key West strain. The cleanup resulted in 797,360,677 cleaned reads, which mapped to 18,840 transcripts of *Ae. aegypti* (Table S1).

**Figure 1:**
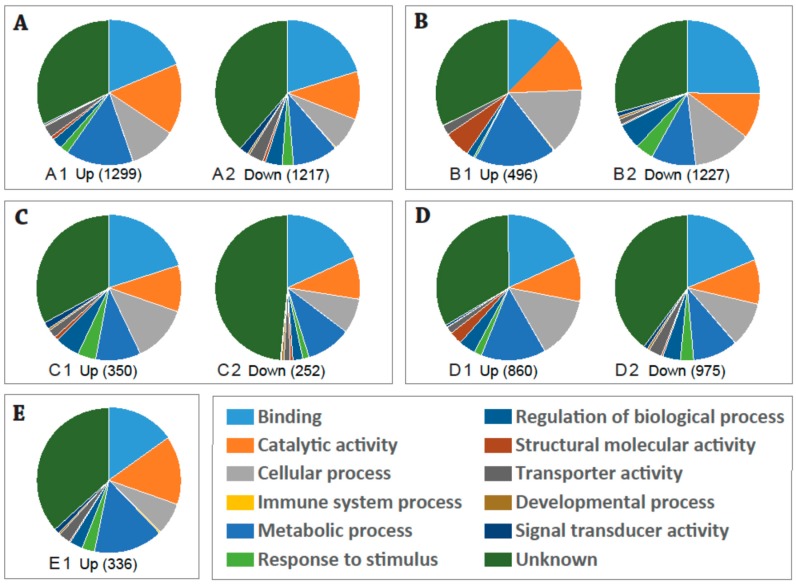
Overview of the functional categories of differentially expressed (DE) transcripts in response to CHIKV infection. DE genes were determined based on statistical analysis by DESeq package. The total number of DE genes for each comparison is shown in parentheses in each figure. Gene Ontology (GO) analysis of DE genes was performed based on the database of AmiGO 2. Up, upregulated DE genes; Down, downregulated DE genes. (**A**) 3 h post infection KW-CHIKV compared with KW-Control, A1 Up and A2 Down; (**B**) 3-day post infection, KW-CHIKV compared with KW-Control, B1 Up and B2 Down; (**C**) 3 days post infection, KW-Control compared with OR-Control, C1 Up and C2 Down; (**D**) 3 days post infection, KW-CHIKV compared with OR-CHIKV, D1 Up and D2 Down; (**E**) 3 days post infection, **E1**: OR-CHIKV compared with OR-Control, E1 UP; OR-CHIKV compared with OR-Control 339 gene down regulated with *p*-adj ≤ 0.01, only three genes were detected in the GO, which are not shown in the figure.

Functional analysis based on Gene Ontology was conducted on the significant differentially expressed (DE) transcripts in response to CHIKV ingestion in the *Ae. aegypti* Key West population/strain. Analysis of mRNA expression profiles of *Ae. aegypti* infected with CHIKV at different time points revealed a relatively high number of DE transcripts 3 h after blood-feeding. There were 2516 DE genes (*p*-adj (*p*-adjust value) ≤ 0.01), including 1299 upregulated and 1217 downregulated genes at 3 h post infection with CHIKV (Figure 1A and Figure S1A). An average of 35% of the transcripts were classified as an unknown function group (35.3% in the total: 32.0% in the Up, 38.7% in the Down). The remaining DE transcripts were matched to the functional categories of Binding (19.4% in the total: 19.0% in the Up, 20% in the Down), Catalytic activity (13.3% in the total: 15.7% in the Up, 10.7% in the Down), Cellular process (9.0% in the total: 10.3% in the Up, 7.5% in the Down), Immune system process (0.11% in the total: 0.4% in the Up, 0.2% in the Down), Metabolic process (12.6% in the total: 15.1% in the Up, 10.0% in the Down), Response to stimulus (2.2% in the total: 1.8% in the Up, 2.6% in the Down), Regulation of biological process (3.0% in the total: 2.4% in the Up, 3.7% in the Down), Structural molecular activity (0.7% in the total: 0.8% in the Up, 0.6% in the Down), Transporter activity (3.0% in the total: 2.6% in the Up, 3.3% in the Down), Developmental process (0.4% in the total: 0.3% in the Up, 0.4% in the Down), and Signal transducer activity (1.1% in the total: 0.3% in the Up, 2.0% in the Down).

Analysis of mRNA expression profiles of *Ae. aegypti* mosquitoes in the Key West strain at different time points of CHIKV infection revealed a relatively high number of DE transcripts 3 days post infection. There were 1723 DE genes (*p*-adj ≤ 0.01, 1227 upregulated and 496 downregulated) in the Key West strain of *Ae. aegypti* at 3 dpi with CHIKV (Figure 1B and Figure S1B). Most of these transcripts (30.7% in the total: 34.3% in the Up, 29.3% in the Down) had unknown functions. The remaining DE transcripts were mainly matched to the functional categories of Binding (21.7% in the total: 13.1% in the Up, 25.1% in the Down), Catalytic activity (10.9% in the total: 12.7% in the Up, 10.1% in the Down), Cellular process (13.7% in the total: 15.7% in the Up, 13.0% in the Down), Immune system process (0.07% in the total: 0.3% in the Up, 0.0% in the Down), Metabolic process (12.6% in the total: 19.2% in the Up, 9.9% in the Down), Response to stimulus (3.0% in the total: 0.5% in the Up, 4.0% in the Down), Regulation of biological process (4.5% in the total: 1.7% in the Up, 5.7% in the Down), Structural molecular activity (2.0% in the total: 6.2% in the Up, 0.3% in the Down), Transporter activity (1.5% in the total: 2.4% in the Up, 1.2% in the Down), Developmental process (0.4% in the total: 0.0% in the Up, 0.06% in the Down), and Signal transducer activity (0.7% in the total: 0.0% in the Up, 0.9% in the Down).

### 2.3. Expression Profiles of the DE Transcripts in Response to Blood-Feeding (Control) between the Key West and Orlando Aedes aegypti Strains

Functional analysis revealed 602 significant DE transcripts (*p*-adj ≤ 0.01) at 3 days post blood infection between the Key West and Orlando strains of *Ae. aegypti*, including 350 upregulated and 252 downregulated genes (Figure 1C and Figure S1C). Most of the DE genes (39.4% in the total: 32.9% in the Up, 48.4% in the Down) were annotated as unknown functions (Figure 1C and Figure S1C). The remaining DE transcripts were categorized into the functional groups of Binding (19.2% in the total: 20.0% in the Up, 18.1% in the Down), Catalytic activity (9.9% in the total: 10.3% in the Up, 9.4% in the Down), Cellular process (10.6% in the total: 12.6% in the Up, 7.8% in the Down), Immune system process (0.0% in the total: 0.0% in the Up, 0.0% in the Down), Metabolic process (10.0% in the total: 10.0% in the Up, 10.0% in the Down), Response to stimulus (3.0% in the average: 4.1% in the Up, 1.4% in the Down), Regulation of biological process (4.1% in the total: 5.5% in the Up, 2.2% in the Down), Structural molecular activity (0.7% in the total: 0.8% in the Up, 0.6% in the Down), Transporter activity (1.7% in the total: 1.9% in the Up, 1.4% in the Down), Developmental process (0.5% in the total: 0.5% in the Up, 0. 6% in the Down), and Signal transducer activity (1.0% in the total: 1.5% in the Up, 0.2% in the Down).

### 2.4. Expression Profiles of the DE Transcripts in Response to CHIKV Infection in the Key West and Orlando Strains of Aedes aegypti

Comparison of the transcriptome profiles of the Key West and Orlando *Ae. aegypti* strains in response to CHIKV 3 dpi revealed 1,835 DE transcripts (*p*-adj ≤ 0.01, 860 upregulated and 975 downregulated, Figure 1D and Figure S1D). Most of the DE transcripts (36.8% in the total: 33.5% in the Up, 39.8% in the Down) had unknown functions (Figure 1D and Figure S1D). The other matched functional categories included binding (18.5% in the total: 18.2% in the Up, 18.7% in the Down), catalytic activity (9.9% in the total: 9.9% in the Up, 9.9% in the Down), cellular process (11.7% in the total: 13.6% in the Up, 9.9% in the Down), immune system process (0.07% in the total: 0.05% in the Up, 0.09% in the Down), metabolic process (12.0% in the total: 14.4% in the Up, 9.9% in the Down), response to stimulus (2.4% in the total: 1.7% in the Up, 3.0% in the Down), regulation of biological process (3.8% in the total: 3.7% in the Up, 3.9% in the Down), structural molecular activity (1.5% in the total: 2.8% in the Up, 0.4% in the Down), transporter activity (2.3% in the total: 1.5% in the Up, 3.0% in the Down), developmental process (0.3% in the total: 0.1% in the Up, 0.5% in the Down), and signal transducer activity (0.7% in the total: 0.5% in the Up, 0.8% in the Down).

### 2.5. Expression Profiles of the DE Transcripts in Response to CHIKV Infection in Orlando Aedes aegypti Strain

Analysis of mRNA expression profiles of the *Ae. aegypti* mosquitoes infected with CHIKV from the Orlando strain detected a relatively high number of DE transcripts 3 days post injection. There were 675 DE genes (*p*-adj ≤ 0.01), including 336 upregulated and 339 downregulated genes (Figure 1E and Figure S1E). Most of these transcripts (99.1% in the total: 18.2% in the Up, 29.3% in the Down) had unknown functions. Among these downregulated genes, only three genes were determined in GO analysis, which was not shown in Figure 1. The remaining DE transcripts mainly matched to the functional categories of binding (7.5% in the total: 15.1% in the Up, 0% in the Down), catalytic activity (7.6% in the total: 15.3% in the Up, 0% in the Down), cellular process (3.5% in the total: 7.0% in the Up, 0.0% in the Down), immune system process (0.2% in the total: 0.4% in the Up, 0.0% in the Down), metabolic process (7.7% in the total: 15.4% in the Up, 0.0% in the Down), response to stimulus (1.4% in the total: 2.9% in the Up, 0.0% in the Down), regulation of biological process (1.4% in the total: 2.9% in the Up, 0.0% in the Down), structural molecular activity (0.2% in the total: 0.4% in the Up, 0.0% in the Down), transporter activity (1.3% in the total: 2.6% in the Up, 0.0% in the Down), developmental process (0.2% in the total: 0.4% in the Up, 0.0% in the Down), and signal transducer activity (0.6% in the total: 1.1% in the Up, 0.0% in the Down).

### 2.6. Changes of the Immune-Related Genes in the Female Adult of the Key West Strain of Ae. aegypti in Response to CHIKV Infection

For the Key West population/strain of *Ae. aegypti,* 3 h post ingestion of CHIKV infected blood a total of 1112 transcripts showed 1.8 or more log2-fold changes (*p*-adj ≤ 0.01, upregulated 456, downregulated 656). Eighty-nine immune-related DE transcripts were significantly upregulated in response to CHIKV after 3 h, compared to the Key West control group. These up- or down-regulated immunity-related genes encoded two allergen, one caspase-1 protein, one cecropin anti-microbial peptide (AAEL000621-RA), five Class B Scavenger Receptor, seven Clip-Domain Serine Protease family B and E, six C-Type Lectin, two fibrinogen and fibronectin, one Gram-Negative Binding Protein, one lachesin, five leucine-rich immune protein, one Leucine-rich repeat-containing protein, two Leucine-rich repeat-containing protein, one SEC14 protein, one serine collagenase 1 precursor, one serine hydroxymethyl transferase, three serine protease, four serine protease inhibitor (serpin), twelve serine-type endopeptidase, four sidestep protein, one signal peptide peptidase, one tep2 protein and one tep3 protein (AAEL008607), one toll protein, two toll-like receptor, twenty-two trypsins, and one venom allergen (Table 2).

When individuals of the Key West strain of *Ae. aegypti* were tested with CHIKV at 3 dpi, a total of 343 DE transcripts showed ≥ ±1.8 log2-fold changes (*p*-adj ≤ 0.01, upregulated 118, downregulated 225). Twenty-five immunity-related DE transcripts were significantly upregulated in response to CHIKV 3 dpi. These up- or down-regulated immunity-related genes encoded one cecropin anti-microbial peptide (AAEL004223), three Clip-Domain Serine Protease family B, three C-Type Lectins (CTL), one defensin anti-microbial peptide, one fibrinogen and fibronectin, one leucine-rich immune protein, three leucine-rich transmembrane protein, one posF21, putative protein, one SEC14 putative protein, one serine/threonine protein kinase, four serine-type endopeptidase, one Trypsin 3A1 Precursor, three trypsin, and one vav1 protein (Table 3).

### 2.7. Changes of the Immune-Related Genes in the Female Adult of the Orlando Strain of Ae. aegypti in Response to CHIKV Infection

When the Orlando strain *Ae. aegypti* were infected with CHIKV at 3 dpi, 316 DE transcripts had ≥±1.8 log2-fold changes (*p*-adj ≤ 0.01, 314 upregulated, 2 downregulated). Thirty-two immunity-related DE transcripts were significantly upregulated in response to CHIKV 3 dpi compared to the Orlando control group. These immunity-related genes encoded two cecropin anti-microbial peptide (AAEL004223 and AAEL017211), one chymotrypsin, two C-Type Lectins (CTL), one C-Type Lysozyme, one defensin anti-microbial peptide, four fibrinogens and fibronectins, one gambicin anti-microbial peptide, one leucine-rich repeat protein, one leucine-rich transmembrane protein, three Serine Protease Inhibitors (serpin), five serine-type endopeptidase, two Trypsin 3A1 Precursor, six trypsin, and two venom allergen (Table 4).

### 2.8. Changes of the Immunity-Related Genes of the Female Adults of Ae. aegypti between the Key West and Orlando Strains in Response to CHIKV Infection

When the Key West strain was compared with the Orlando strain among individuals following 3 dpi with CHIKV, a total of 856 transcripts had 1.8 log2-fold or above changes (*p*-adj ≤ 0.01, upregulated 258, downregulated 598). Thirty-three immunity-related DE transcripts were significantly up/down-regulated in response to CHIKV 3 dpi when Key West CHIKV-infected mosquitoes were compared to Orlando CHIKV-infected individuals. These up- or down-regulated immunity-related genes encoded two allergen, one br serine/threonine-protein kinase, one cecropin anti-microbial peptide (AAEL017211), three Class B Scavenger Receptors, one Clip-Domain Serine Protease family B, two C-Type Lectins (CTL), one D7 protein, three fibrinogen and fibronectin, one Gram-Negative Binding Protein, one lachesin, one leucine-rich repeat protein, two leucine-rich transmembrane protein, three Serine Protease Inhibitor (serpin), one serine protease, one serine-pyruvate aminotransferase, one serine-type endopeptidase, one TOLL pathway signaling, one toll protein, two Toll-like receptor, two trypsins, and two venom allergens (Table 5).

### 2.9. Immunity-Related Genes of the Ae. aegypti Female Adult between the Key West and Orlando Strains in Response to Ingestion of Blood

When mosquitoes of the Key West strain were compared with the Orlando strain of *Ae. aegypti* 3 dpi, a total of 414 transcripts had 1.8 or above log2-fold changes (*p*-adj ≤ 0.01, upregulated 219, downregulated 195). Thirteen immunity-related DE transcripts in the Key West strain were significantly regulated in response to ingestion of the CHIKV-infected blood at 3 dpi compared with the Orlando strain. These up- or down-regulated immunity-related genes encoded one autophagy-related gene, one fibrinogen and fibronectin, one Gram-Negative Binding Protein, one JAKSTAT pathway signaling Signal, one protein serine/threonine kinase, one scavenger receptor, one serine protease, one serine/threonine protein kinase, two serine-type endopeptidases, two trypsins, and one viral IAP-associated factor (Table 6).

### 2.10. AaeDEFA, AaeDEFD, AaeDEFa, AaeDNR1, AaeCECH, and AaeTEP3 Transcriptional Induction Following Ingestion of CHIKV-Infected Blood in Ae. aegypti Females

**Figure 2:**
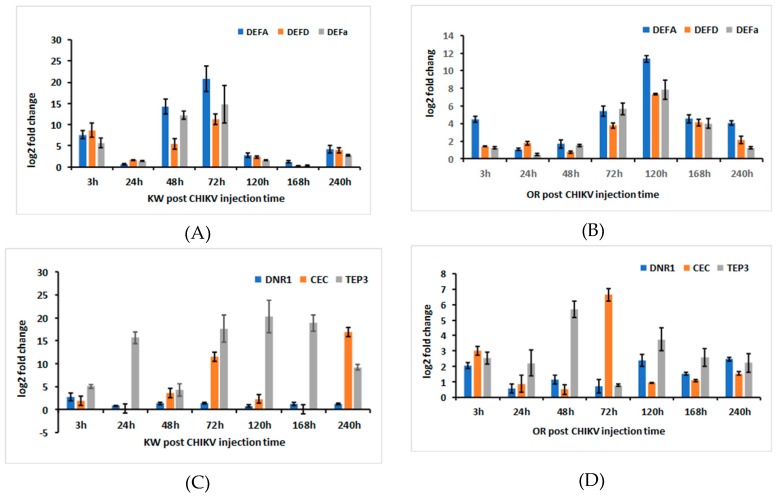
AaeDEFA, AaeDEFD, AaeDEFa, AaeDNR1, AaeCECH and AaeTEP3 relative expression level fold-changes in *Aedes aegypti* females that ingested CHIKV infected blood. The fold change was calculated using the 2^[−average ΔΔ*C*T]^ method. Δ*C*t (Control) = Ct (AaeDEFA/ AaeDEFD/AaeDEFa /AaeDNR1/AaeCECH/AaeTEP3) − Ct (AeaActin); ΔCt (infected-CHIKV) = Ct (AaeDEFA/ AaeDEFD/AaeDEFa /AaeDNR1/AaeCECH/AaeTEP3) − Ct (AeaActin); ΔΔCt =ΔCt (infected-CHIKV) − ΔCt (Control). The 3, 24, 48, 72, 120, 168, and 240 h (hours) represented gene expression post infection with CHIKV, (**A**,**C**) KW strain female *Ae. Aegypti*, (**B**,**D**) Orlando strain female *Ae. aegypti*.

To characterize *Ae. aegypti* defensin and other immune-related genes in response to CHIKV exposure, we measured *AaeDEFA, AaeDEFD,* *AaeDEFa, AaeDNR1, AaeCECH,* and *AaeTEP3* expressions in orally ingested *Ae aegypti*. Multivariate analysis of variance (MANOVA) showed the significant effects of strain of *Ae. aegypti*, time, and their interaction. For the significant strain effect, standardized canonical coefficients (SCCs) showed that *AaeTEP3* was the primary contributor with approximately 2–16-fold greater effect than all other genes (Table 7). SCCs showed that *AaeDEFA* had the second highest contribution to the significant strain effect with approximately 1.3–8-fold greater effect than the other genes (Table 7). SCCs showed that *AaeDEFa* had the third highest contribution to the significant strain effect with about 3–6-fold greater effect than the other genes (Table 7). Expression of *AaeDEFa* was negatively correlated with expression of *AaeTEP3* and *AaeDEFA.* Differential expression of *AaeTEP3, AaeDEFa,* and *AaeDEFa* were all much higher for the Key West than Orlando strain of *Ae. aegypti* (Figure 2A–D).

For both the significant time and interaction effects, SCCs showed that *AaeDEFA* was the primary contributor (time, 2–11-fold, interaction, 2–44-fold) with *AaeDEFD* (time, 2–6-fold, interaction, 2–24-fold) and *AaeTEP3* (time, 2–6-fold, interaction, 2–20-fold) having similar and the second highest contribution to the significant time and interaction effects. SCCs showed that *AaeCECH* was the third highest contributor (time, 2–4-fold, interaction, 2–13-fold) to the significant time and interaction effects. For the time effect, *AaeTEP3* was negatively correlated with the expression of these three other genes. For the interaction, *AaeCECH* was negatively correlated with the expression of these three other genes (Table 7).

Because the interaction was significant, we focused on pairwise comparisons of strain by time combinations. Specifically, we compared the two strains of *Ae. aegypti* at each distinct time period (e.g., Key West versus Orlando at 3 h post infection), resulting in less than all possible comparisons. Gene expression of *AaeDEFA* was significantly different between the two strains of *Ae. aegypti* except at 24 and 240 h post infection. Gene expression of *AaeDEFA* was 2–9-fold significantly higher for the Key West than the Orlando strains of *Ae. aegypti* at most time points, except 120 and 168 h, where Orlando was 4-fold significantly higher. The general pattern of expression of *AaeDEFA* was characterized by an increase from 3 h post infection to the highest expression at 72 h for Key West and 120 h for Orlando, followed by a sharp decline in expression at later periods for both strains of *Ae. aegypti* (Figure 2A,B). Gene expression of *AaeDEFD* was 2–7-fold significantly higher for the Key West than the Orlando strain of *Ae. aegypti* at 3, 48, 72, and 240 h post infection. Gene expression of *AaeDEFD* was 3–15-fold significantly higher for the Orlando than the Key West strain of *Ae. aegypti* at 120 h and 168 h post infection. The two strains did not differ in expression of *AaeDEFD* at 24 h post infection. The highest gene expression of *AaeDEFD* was observed 72 h and 120 h post ingestion for the Key West and the Orlando strains, respectively. Gene expression of *AaeDEFD* declined after these periods for both *Ae. aegypti* strains (Figure 2A,B). Similarly, gene expression of *AaeTEP3* was 4–22-fold significantly higher for the Key West than the Orlando strain of *Ae. aegypti* at all times, except 3 and 48 h post ingestion (Figure 2C,D). Gene expression of *AaeCECH* was similar for both *Ae. aegypti* strains at most time points. However, *AaeCECH* was 2–11-fold significantly higher for the Key West than the Orlando strains of *Ae. aegypti* at 48, 72, and 240 h post ingestion (Figure 2C,D). In summary, *AaeDEFA, AaeDEFD, AaeDEFa, AaeDNR1, AaeCECH,* and *AaeTEP3* expressions were significantly higher for most times post ingestion for the Key West strain, except for 120 and 168 h post ingestion when expression of *AaeDEFA* and *AaeDEF* was higher for the Orlando strain of orally infected *Ae. aegypti.*

## 3. Discussion

Understanding the *Aedes* vector–chikungunya virus interactions of natural and geographically distinct populations is fundamentally important since it may enable the search for new methods and strategies for interrupting arbovirus transmission. Previous transcriptomic studies have shown altered pathways in response to viral infection between genetically distinct strains of mosquito species [29,30]. In the current study, transcripts enriched in CHIKV infected females encoded proteins associated with functions which are largely unknown, binding catalytic activities, cellular process, metabolism process, response to stimulus, regulation of biological process, and immune system process (Figure 1 and Figure S1). A previous study disclosed that serine-type endopeptidases and trypsins were significantly upregulated in mosquitoes following CHIKV ingestion [17]. Our transcriptomic analysis also showed that serine-type endopeptidases and trypsins were significantly upregulated in *Ae. aegypti* following CHIKV 3 days post infection in the Key West strain and Orlando strains. Comparison between the Key West and Orlando strains 3 days post infection with CHIKV showed that both serine-type endopeptidases and trypsins were significantly downregulated.

Numerous reports have shown altered expression of cecropin anti-microbial peptide (CECH) in response to exposure to pathogens, including arboviruses [36,37,51,52,53]. Comparison of the transcriptome profiles showed that immunity related genes, including cecropin anti-microbial peptides (CECH) (AAEL000621-RA, AAEL004223-RA, and AAEL017211-RA) were significantly upregulated in the Key West strain *Ae. aegypti* at two time points (3 h and 3 days) post infection with CHIKV compared with Control of the same strain, and also significantly upregulated in the Orlando strain *Ae. aegypti* 3 days post infection with CHIKV compared with Control of the same strain. However, *AaeCECH* was significantly downregulated in the Key West strain *Ae. aegypti* 3 days post infection with CHIKV compared with the Orlando strain *Ae. aegypti* (Table 5). *AaeCECH* was not significantly downregulated in the Key West strain *Ae. aegypti* 3 days Control compared with Control in the Orlando strain (Table 6). Transcriptomic analysis and q-PCR data showed that *AaeCECH* gene were highly expressed in the Key West strain and the Orlando strain in response to the CHIKV infection (Table 2, Table 3 and Table 4, Figure 2C,D). These data may implicate that *AaeCECH* genes are important for CHIKV infection response, and by extension other arboviruses, in the mosquitoes.

The data presented here represent the first transcriptomic analysis of immune-related genes from a field population of *Ae. aegypti* and provide useful information for future investigations that aim to elucidate interactions between mosquito vector and arboviruses. Host-virus interactions are representative of complex coevolved adaptations. These dynamic interactions are convincingly demonstrated in the activity of transcriptomes in mosquito hemocytes that comprise the immune system in invertebrates. Designing primers and further examining the innate immune-related genes will provide more information underlying antiviral activity in mosquitoes that limit viral infection. Understanding the mechanism of mosquito–virus interactions, including antiviral defense, and identifying the immune evasion strategies of pathogens will aid in the development of methods to interrupt transmission of arboviruses through the manipulation of mosquitoes.

## 4. Materials and Methods

### 4.1. Aedes aegypti 

Two lines of *Ae. aegypti* from Florida were used in this study. A wild-type line (strain) of *Ae. aegypti* originated from larvae collected in Key West (24.55° N, 81.78° W), Florida (FL), USA, in 2014. The Key West strain of *Ae. aegypti*, referred to as the natural population (F4 generation used for experiments), was maintained at the Florida Medical Entomology Laboratory (FMEL) in Vero Beach, FL, USA. A long-standing laboratory strain of *Ae. aegypti* was collected from Orlando, FL, USA, in 1952 and reared in the Mosquito and Fly Research Unit, Center for Medical, Agricultural and Veterinary Entomology, ARS-USDA in Gainesville, FL, USA.

### 4.2. Chikungunya Virus Infection

Virus stocks of Chikungunya from Réunion Island (Indian Ocean lineage, LR2006-OPY1, GenBank accession: KT449801) were cultured at 37 °C and 5% carbon dioxide in Vero (African green monkey kidney) cells with media (M199 medium supplemented with 10% fetal bovine serum, penicillin/streptomycin, and myostatin) for an incubation of three days. Following incubation, supernatants from infected cell lines were collected and combined with defibrinated bovine blood and ATP (0.005 M) and presented to four-day-old female mosquitoes for oral feeding using a membrane system (Hemotek, Lancashire, UK). Control blood meals were prepared similarly except that monolayers of Vero cells were inoculated with media only. Samples of infected blood were taken at the time of the feedings for determination of virus titer. Mosquitoes were fed 8.0–8.3 log10 pfu/mL of CHIKV. Specific procedures are described in previous studies [29,35,39].

Blood-engorged mosquitoes were sorted using light microscopy (10×) and transferred to cages (h by d: 10 cm by 10 cm) and incubated at 30 °C and a 12 h light/dark cycle. During incubation, mosquitoes were allowed continuous access to a 10% sucrose solution on cotton pads. Each cohort of mosquitoes in the cages were provided with an oviposition substrate. Mosquitoes 3, 24, 48, 72, 120, 168, and 240 h post feeding were stored at −80 °C for later testing. Separate cohorts of mosquitoes were tested for infection and viral titer on 3, 7, and 10 days following ingestion of CHIKV-infected blood.

### 4.3. RNA Extraction

Samples (10 mosquitoes pool for each sample and three replicates for each time point) were homogenized with a plastic pestle in a 1.5 mL tubes. Total RNAs were extracted using the TRIzol reagent according to the manufacturer’s instruction (Ambion, Life Technologies, Carlsbad, CA, USA) and followed the standard protocol [35,39]. The RNA samples were digested by DNase I (RNase-free), according to the manufacturer’s instructions (Thermo Scientific, Wilmington, DE, USA). The purified RNA samples were quantitated by NANODROP 2000 Spectrophotometer (Thermo Scientific, Wilmington, DE, USA).

### 4.4. RNA-Sequencing Library Construction and Sequencing

The RNA-sequencing libraries were constructed at ICBR Gene Expression and Genotyping using NEBNext® Ultra™ Directional RNA Library Prep Kit for Illumina (NEB, Ipswich, MA, USA), following manufacturer’s recommendations. Basically, 500 ng of high-quality total RNA (RIN ≥ 7) was used for mRNA isolation using NEBNext Ploy(A) mRNA Magnetic Isolation module (New England Biolabs, Ipswich, MA, USA). This was then followed by RNA library construction with NEBNext Ultra II Directional Lib Prep (New England Biolabs, Ipswich, MA, USA), according to the manufacturer’s user guide. Briefly, RNA is fragmented in NEBNext First Strand Synthesis Buffer by heating at 94 °C for the desired time. This step is followed by first strand cDNA synthesis using reverse transcriptase and oligo dT primers. Synthesis of ds cDNA is performed using the 2nd strand master mix provided in the kit, followed by end-repair and adaptor ligation. Finally, the library is enriched (each library has a unique barcode) by 11 cycles of amplification, and purified by Agencourt AMPure beads (Beckman Coulter, Atlanta, GA, USA, catalog # A63881). Finally, each 14 individual libraries were pooled (total of 3 pools) with equimolar and sequenced by Illumina HiSeq 3000 2X 100 cycles run for total of 1 run (Illumina Inc., San Diego, CA, USA).

### 4.5. RNA-sequencing Data Analysis

Raw reads generated by the Illumina Hiseq 3000 were processed with the Cutadapt (Martin 2011). All partial sequencing adaptors, low-quality bases (phred-like score < 20), short reads (<40 bases), and potential errors from sequencing and library construction were trimmed off or removed. The processed paired-end reads were mapped against the 18,840 transcripts of *Ae. aegypti* from the VectorBase (https://www.vectorbase.org/organisms/aedes-aegypti/liverpool) by using the bowtie2 mapper (v. 2.3.4.3). The mapping results were further analyzed with samtools and the scripts developed in house at ICBR to remove potential PCR duplicates. Principal component analysis (PCA) was used for quality control, identifying problems with experimental design, mislabeled samples, and to visualize variation between expression analysis samples. The gene expression levels were assessed by counting the number of mapped reads for each transcript [54]. Significantly up- and down-regulated genes were selected using the adjusted *p*-value, and fold-change for downstream analysis.

### 4.6. Assignments of Gene Ontology (GO) Terms and Pathway Analyses

The GO enrichment analysis (http://amigo.geneontology.org/amigo) were based on the GO terms of *Ae. aegypti* genes from the VectorBase. The selected genes (*p*-adj ≤ 0.01) were grouped as the downregulated and upregulated gene pools based on the log2 transformed-fold-change of the RNA-sequencing results, and then assigned to the GO hierarchies and functional categories, including immune system process (GO:0002376), binding (GO:0005488), catalytic activity (GO:0003824), cellular process (GO:0009987), metabolic process (GO:0008152), response to stimulus (GO:0050896), regulation of biological process (GO:0050789), structural molecular activity (GO:0005198), transporter activity (GO:0005215), developmental process (GO:0032502), and signal transducer activity (GO:0004871). The genes that were not assigned GO terms or categorized to other functional groups were assigned as the unknown group.

### 4.7. cDNA Synthesized and qPCR Amplification

cDNAs from 2 µg of total purified RNA were synthesized using a Cloned AMV First-Strand cDNA Synthesis Kit Invitrogen™ and Oligo (dT)20 primer, according to the manufacturer’s instructions (Invitrogen, Carlsbad, CA, USA). The reaction was terminated by heat inactivation at 95 °C for 5 min. The cDNA samples for qPCR from infected treatment and controls were diluted by adding 80 µL ddH_2_O to 20 µL reaction solution [55,56].

The quantitative PCR (qPCR) assay for target genes *AaeDEFA*, *AaeDEFD, AaeDEFa, AeaCECH, AaeTEP3,* and *AaeDNR1,* and reference gene *AaeActin* in *Ae. aegypti* was accomplished using a BIO-RAD C1000 Touch Thermal Cycler, CFX 96™ Real-Time System (BIO-RAD, Hercules, CA, USA). The qPCR reaction mixture with a volume of 15 µL in a Multiwell Plates 96 contained 1 µL diluted cDNA, 0.5 µM primers and 1X master mix of PowerUP SYBR® Green Master Mix (Applied Biosystems, Thermo Fisher Scientific, Foster City, CA, USA). In every qPCR run, *AaeActin* was employed as an internal control to normalize for variation in the amount of cDNA template. The PCR primers for *AaeDEFA,* and other genes were designed from the coding region based on GenBank, Accession Number using Primer3 (http://primer3.ut.ee) (Table 8). The qPCR thermal cycling parameters were the same as in the previous publication [56]. Relative expression levels were calculated as follows for the developmental stages. First, *AaeDEFA* transcript levels relative to a standard (*AaeActin*) were calculated using the formula *Δ*CT = CT (*AaeDEFA*) − CT (*AaeActin*). Then, *ΔΔ*CT= *Δ*CT (infected) − *Δ*CT (control) value for each sample was calculated. Third, relative expression levels were calculated using the equation 1 × 2^[−average*ΔΔC*T]^ [35,56,57,58,59].

### 4.8. Statistical Analysis

Multivariate analysis of variance (MANOVA) and ANOVA were used to measure the gene expression of *AaeDEFA, AaeDEFD, AaeDEFa, AeaCECH, AaeTEP3,* and *AaeDNR1* following ingestion of CHIKV-infected blood. The relative contribution and relationship of *AaeDEFA, AaeDEFD, AaeDEFa, AeaCECH, AaeTEP3,* and *AaeDNR1* to treatment effects were assessed using standardized canonical coefficients (SCC) (PROC GLM, SAS 9.22). When significant effects were detected, we used univariate comparisons among treatment least-squares means (Tukey–Kramer method). Differential gene expression of all treatment factors (mosquito strain, time) and their interactions was evaluated. A Pearson product–moment correlation coefficient was used to measure the strength of the linear relationship between variables RNA-sequencing and qPCR data sets. The analysis showed a strong positive relationship between the two variables (*r* = 0.85, *p* = 0.0035).

## Figures and Tables

**Table 1:** Chikungunya virus (CHIKV) titers (log10 pfu/mL) (Least Squares means ± Standard Error) in infectious blood meals and mosquitoes of the Key West and Orlando strains of *Aedes aegypti*. Titers followed by the same letter are not significantly different from one another. Comparisons of time points are presented only for Key West (lower case letters) and Orlando (upper case letters) strains.

Strains	Initial Dose in Bloodmeal	Freshly Fed	1 Day Post Infection	2 Days Post Infection	5 Days Post Infection	7 Days Post Infection	10 Days Post Infection
**Key West**	8.0 ± 0.09	4.89 ± 0.39	3.79 ± 0.23 a	4.47 ± 0.32 ab	5.87 ± 0.39 bc	5.83 ± 0.39 bc	4.96 ± 0.39 ab
**Orlando**	8.3 ± 0.08	5.68 ± 0.39	3.09 ± 0.23 A	1.70 ± 0.30 B	4.78 ± 0.39 C	4.02 ± 0.32 AC	4.24 ± 0.39 AC

CHIKV (La Réunion strain LR2006-OPY1, GenBank KT449801) from a human infected on La Réunion Island in 2006 [50]. Primers: CHIKV-FWD: 5′GTACGGAAGGTAAACTGGTATGG-3’; CHIKV-REV: 5′-TCCACCTCCCACTCCTTAAT-3′.

**Table 2:** Immune-related genes that were significantly regulated in the Key West strain of *Aedes aegypti* 3 h post infection with CHIKV compared with Control in Key West strain (*p*-adj ≤ 0.01, log2-fold change ≥ ± 1.8).

Transcript ID	Log2 FC	*p*-adj	Gene Description
AAEL004199-RA	−1.9388	3.4 × 10^−4^	allergen
AAEL003057-RB	2.8963	4.2 × 10^−6^	allergen
AAEL014658-RA	2.2755	4.4 × 10^−4^	caspase-1 protein
AAEL000621-RA	2.2216	3.4 × 10^−4^	cecropin anti-microbial peptide
AAEL005374-RA	−3.2830	1.1 × 10^−27^	Class B Scavenger Receptor (CD36 domain)
AAEL005979-RA	−2.0366	1.9 × 10^−4^	Class B Scavenger Receptor (CD36 domain)
AAEL005981-RA	−1.8895	1.5 × 10^−5^	Class B Scavenger Receptor (CD36 domain)
AAEL000234-RA	1.8135	1.6 × 10^−4^	Class B Scavenger Receptor (CD36 domain)
AAEL009432-RA	4.0915	3.5 × 10^−12^	Class B Scavenger Receptor (CD36 domain)
AAEL001098-RA	−2.2546	2.9 × 10^−7^	Clip-Domain Serine Protease
AAEL014137-RA	−2.3522	3.2 × 10^−7^	Clip-Domain Serine Protease family B
AAEL008668-RA	1.8083	1.8 × 10^−3^	Clip-Domain Serine Protease family B
AAEL000760-RA	2.2343	4.1 × 10^−5^	Clip-Domain Serine Protease family B
AAEL005648-RA	−1.9389	2.0 × 10^−3^	Clip-Domain Serine Protease family B
AAEL005792-RA	−2.0842	5.1 × 10^−4^	Clip-Domain Serine Protease family E
AAEL001077-RA	−1.9692	6.8 × 10^−13^	Clip-Domain Serine Protease family B
AAEL008299-RA	−2.4009	7.4 × 10^−5^	C-Type Lectin (CTL)
AAEL004679-RA	−2.0008	5.5 × 10^−3^	C-Type Lectin (CTL)
AAEL000533-RA	2.0222	1.9 × 10^−4^	C-Type Lectin (CTL)
AAEL000556-RA	3.3807	2.4 × 10^−28^	C-Type Lectin (CTL)
AAEL011078-RA	−2.5784	1.9 × 10^−3^	C-Type Lectin (CTL)—galactose binding
AAEL011455-RA	2.3166	2.9 × 10^−4^	C-Type Lectin (CTL)—mannose binding
AAEL009384-RA	−1.9456	3.7 × 10^−7^	fibrinogen and fibronectin
AAEL000726-RA	3.6985	4.1 × 10^−42^	fibrinogen and fibronectin
AAEL003894-RA	−1.8649	3.0 × 10^−3^	Gram-Negative Binding Protein (GNBP)
AAEL003966-RA	−1.9430	2.8 × 10^−6^	lachesin
AAEL010125-RA	2.3599	8.2 × 10^−10^	leucine-rich immune protein (Coil-less)
AAEL010132-RA	2.4747	2.5 × 10^−5^	leucine-rich immune protein (Long)
AAEL012255-RA	1.8045	7.8 × 10^−4^	leucine-rich immune protein (Short)
AAEL001420-RA	1.9602	5.0 × 10^−10^	leucine-rich immune protein (Short)
AAEL001401-RA	2.0463	3.8 × 10^−18^	leucine-rich immune protein (Short)
AAEL010772-RA	−2.2935	3.6 × 10^−6^	leucine-rich repeat-containing protein
AAEL011760-RA	−2.2672	9.7 × 10^−4^	leucine-rich transmembrane protein
AAEL012093-RA	2.0559	2.0 × 10^−4^	leucine-rich transmembrane protein
AAEL008271-RA	6.5329	4.5 × 10^−12^	SEC14, putative protein
AAEL007432-RA	6.4799	1.5 × 10^−65^	serine collagenase 1 precursor
AAEL002510−RB	1.8323	1.4 × 10^−4^	serine hydro×ymethyltransferase
AAEL000224-RA	−3.2320	2.1 × 10^−3^	serine protease
AAEL013427-RA	−3.1475	4.8 × 10^−4^	serine protease
AAEL007106-RA	2.1779	2.0 × 10^−4^	serine protease
AAEL002704-RB	3.1322	3.4 × 10^−37^	Serine Protease Inhibitor (serpin)
AAEL002731-RA	2.1521	2.7 × 10^−10^	Serine Protease Inhibitor (serpin)
AAEL007420-RB	3.3676	6.7 × 10^−23^	Serine Protease Inhibitor (serpin)
AAEL003182-RA	3.5677	2.1 × 10^−43^	Serine Protease Inhibitor (serpin)
AAEL011891-RA	2.7316	6.5 × 10^−3^	serine-type endopeptidase
AAEL006902-RA	3.1972	6.8 × 10^−6^	serine-type endopeptidase
AAEL008567-RA	3.5171	5.4 × 10^−13^	serine-type endopeptidase
AAEL001703-RA	3.7862	2.8 × 10^−8^	serine-type endopeptidase
AAEL008784-RA	3.9399	6.6 × 10^−6^	serine-type endopeptidase
AAEL001674-RA	5.4452	6.4 × 10^−6^	serine-type endopeptidase
AAEL014188-RA	5.5757	1.8 × 10^−9^	serine-type endopeptidase
AAEL009843-RA	5.7334	2.4 × 10^−59^	serine-type endopeptidase
AAEL013284-RA	5.8404	1.4 × 10^−8^	serine-type endopeptidase
AAEL003060-RA	6.2886	7.2 × 10^−129^	serine-type endopeptidase
AAEL001693-RA	7.8599	1.2 × 10^−109^	serine-type endopeptidase
AAEL001690-RA	8.1855	7.1 × 10^−114^	serine-type endopeptidase
AAEL008236-RA	−2.6394	9.4 × 10^−3^	sidestep protein
AAEL000398-RA	−2.4779	4.8 × 10^−3^	sidestep protein
AAEL009943-RA	−2.2813	5.3 × 10^−5^	sidestep protein
AAEL010645-RA	3.8037	9.9 × 10^−3^	sidestep protein
AAEL011989-RB	1.9061	4.9 × 10^−10^	signal peptide peptidase
AAEL014755-RA	−2.4382	1.7 × 10^−3^	tep2 protein
AAEL008607-RA	−1.8312	4.5 × 10^−10^	tep3 protein
AAEL015018-RA	2.2916	6.7 × 10^−4^	toll protein
AAEL004000-RA	−2.9297	5.8 × 10^−4^	Toll-like receptor
AAEL009551-RA	−2.8670	1.4 × 10^−3^	Toll-like receptor
AAEL012780-RA	−5.2801	3.1 × 10^−3^	trypsin
AAEL006414-RA	−2.6981	1.7 × 10^−7^	trypsin
AAEL006430-RA	−2.6562	2.2 × 10^−3^	trypsin
AAEL006429-RA	−2.4442	3.8 × 10^−5^	trypsin
AAEL005764-RA	−2.1564	7.9 × 10^−16^	trypsin
AAEL010202-RA	1.9991	2.3 × 10^−3^	trypsin
AAEL008093-RA	2.6642	1.1 × 10^−7^	trypsin
AAEL008085-RA	4.4810	5.5 × 10^−27^	trypsin
AAEL013703-RA	5.2463	1.2 × 10^−72^	trypsin
AAEL006425-RA	5.2958	2.4 × 10^−19^	trypsin
AAEL013715-RA	8.8303	1.9 × 10^−42^	trypsin
AAEL007601-RA	9.5930	1.2 × 10^−90^	trypsin
AAEL013707-RA	9.8853	1.9 × 10^−50^	trypsin
AAEL013714-RA	10.627	4.3 × 10^−48^	trypsin
AAEL010196-RA	10.743	1.5 × 10^−36^	trypsin
AAEL007818-RB	2.8577	9.9 × 10^−7^	Trypsin 3A1 Precursor
AAEL013712-RA	6.7673	1.2 × 10^−24^	Trypsin 5G1 Precursor
AAEL013629-RA	3.3504	2.1 × 10^−13^	trypsin-alpha
AAEL008079-RA	6.2776	1.7 × 10^−16^	trypsin-alpha
AAEL006403-RA	−3.7628	4.8 × 10^−10^	trypsin-beta
AAEL008080-RA	2.3952	1.2 × 10^−5^	trypsin-eta
AAEL013628-RA	4.8704	4.2 × 10^−14^	trypsin-eta
AAEL000793-RA	3.3944	1.1 × 10^−48^	venom allergen

**Table 3:** Immune-related genes that were significantly regulated in the Key West strain of *Aedes aegypti* 3 days post ingestion with CHIKV compared with Control in Key West strain (*p*-adj ≤ 0.01, log2-fold change ≥ ± 1.8)**.**

Transcript ID	Log2 FC	*p*-adj	Gene Description
AAEL004223-RA	1.8947	1.1 × 10^−6^	cecropin anti-microbial peptide
AAEL000037-RA	3.0672	1.4 × 10^−3^	Clip-Domain Serine Protease family B
AAEL006161-RB	3.1447	1.1 × 10^−3^	Clip-Domain Serine Protease family B
AAEL003632-RA	3.0778	6.0 × 10^−3^	Clip-Domain Serine Protease family B
AAEL005641-RA	3.2355	4.9 × 10^−4^	C-Type Lectin (CTL)—galactose binding
AAEL011455-RA	4.1030	4.2 × 10^−3^	C-Type Lectin (CTL)—mannose binding
AAEL012353-RA	4.3829	1.3 × 10^−3^	C-Type Lectin (CTL)
AAEL003857-RA	7.3899	4.1 × 10^−6^	defensin anti-microbial peptide
AAEL006691-RA	1.9004	8.2 × 10^−3^	fibrinogen and fibronectin
AAEL001401-RA	2.7153	3.9 × 10^−4^	leucine-rich immune protein (Short)
AAEL005734-RA	−1.9928	2.9 × 10^−14^	leucine-rich transmembrane protein
AAEL002295-RA	2.2469	6.8 × 10^−3^	leucine-rich transmembrane protein
AAEL003597-RB	2.4398	1.6 × 10^−3^	leucine-rich transmembrane protein
AAEL005844-RA	−2.2151	9.8 × 10^−25^	posF21, putative protein
AAEL008271-RA	3.0341	8.6 × 10^−3^	SEC14 putative protein
AAEL003896-RA	−1.7801	2.4 × 10^−27^	serine/threonine protein kinase
AAEL009244-RA	1.8418	2.3 × 10^−7^	serine-type endopeptidase
AAEL001703-RA	2.0534	2.9 × 10^−4^	serine-type endopeptidase
AAEL009843-RA	2.8570	8.1 × 10^−3^	serine-type endopeptidase
AAEL003060-RA	2.3460	1.1 × 10^−5^	serine-type endopeptidase
AAEL007818-RB	2.5953	7.1 × 10^−4^	Trypsin 3A1 Precursor
AAEL006425-RA	2.3896	3.0 × 10^−9^	trypsin
AAEL013703-RA	2.5507	3.3 × 10^−4^	trypsin
AAEL008080-RA	2.7631	2.1 × 10^−4^	trypsin-eta
AAEL012473-RB	−2.1156	9.4 × 10^−6^	vav1 protein

**Table 4:** Immunity-related genes significantly regulated in the Orlando strain *Aedes aegypti* 3 days post infection with CHIKV compared with Control in Orlando strain (*p*-adj ≤ 0.01, log2-fold change ≥ ± 1.8).

Transcript ID	Log2 FC	*p*-adj	Gene Description
AAEL004223-RA	2.3577	4.4 × 10^−5^	cecropin anti-microbial peptide
AAEL017211-RA	2.3359	3.3 × 10^−4^	cecropin anti-microbial peptide
AAEL009680-RB	4.7006	1.6 × 10^−10^	chymotrypsin
AAEL000556-RA	4.0712	6.3 × 10^−6^	C-Type Lectin (CTL)
AAEL000533-RA	3.0535	9.0 × 10^−4^	C-Type Lectin (CTL)
AAEL009670-RA	3.6210	1.3 × 10^−3^	C-Type Lysozyme (Lys-D)
AAEL003849-RA	3.1292	4.3 × 10^−3^	defensin anti-microbial peptide
AAEL000726-RA	4.7815	5.9 × 10^−8^	fibrinogen and fibronectin
AAEL006691-RA	3.2048	2.7 × 10^−6^	fibrinogen and fibronectin
AAEL007942-RA	3.4734	7.6 × 10^−6^	fibrinogen and fibronectin
AAEL011007-RA	4.5979	1.0 × 10^−6^	fibrinogen and fibronectin
AAEL004522-RA	3.1695	2.6 × 10^−3^	gambicin anti-microbial peptide
AAEL012092-RA	2.0567	8.4 × 10^−3^	leucine-rich repeat
AAEL002295-RA	2.6054	1.3 × 10^−4^	leucine-rich transmembrane protein
AAEL007420-RB	5.1106	2.4 × 10^−5^	Serine Protease Inhibitor (serpin)
AAEL003182-RA	4.6609	7.4 × 10^−4^	Serine Protease Inhibitor (serpin)
AAEL002704-RB	4.2747	1.5 × 10^−3^	Serine Protease Inhibitor (serpin)
AAEL006902-RA	3.6563	3.6 × 10^−6^	serine-type endopeptidase
AAEL008567-RA	3.1476	7.6 × 10^−9^	serine-type endopeptidase
AAEL008784-RA	3.6928	1.4 × 10^−4^	serine-type endopeptidase
AAEL009244-RA	3.0981	4.5 × 10^−3^	serine-type endopeptidase
AAEL003060-RA	2.2077	3.1 × 10^−4^	serine-type endopeptidase
AAEL007818-RB	4.8704	5.5 × 10^−3^	Trypsin 3A1 Precursor
AAEL007818-RA	4.1455	3.2 × 10^−5^	Trypsin 3A1 Precursor
AAEL006425-RA	3.2268	1.5 × 10^−10^	trypsin
AAEL008085-RA	2.7895	2.4 × 10^−4^	trypsin
AAEL013623-RA	3.0234	9.7 × 10^−4^	trypsin
AAEL013703-RA	2.7960	7.2 × 10^−13^	trypsin
AAEL008079-RA	2.9739	1.9 × 10^−10^	trypsin-alpha
AAEL013628-RA	4.2736	7.2 × 10^−13^	trypsin-eta
AAEL000793-RA	4.7662	6.8 × 10^−5^	venom allergen
AAEL002693-RA	2.4160	9.6 × 10^−3^	venom allergen

**Table 5:** Immunity-related genes significantly regulated in the Key West strain *Aedes aegypti* 3 days post infection with CHIKV compared with Orlando strain *Aedes aegypti* (*p*-adj ≤ 0.01, log2-fold change ≥±1.8)**.**

Transcript ID	Log2 FC	*p*-adj	Gene Description
AAEL010235-RA	−5.6356	4.0 × 10^−18^	allergen
AAEL006424-RA	−3.1799	3.1 × 10^−4^	allergen
AAEL001139-RA	−3.7087	9.1 × 10^−3^	br serine/threonine-protein kinase
AAEL017211-RA	−2.0884	1.4 × 10^−4^	cecropin anti-microbial peptide
AAEL005374-RA	−3.5005	2.6 × 10^−4^	Class B Scavenger Receptor (CD36 domain)
AAEL000234-RA	−2.1910	2.4 × 10^−3^	Class B Scavenger Receptor (CD36 domain)
AAEL006355-RA	2.9459	5.5 × 10^−5^	Class B Scavenger Receptor
AAEL003628-RA	2.5283	2.5 × 10^−4^	Clip-Domain Serine Protease family B
AAEL011612-RB	−3.1179	1.0 × 10^−2^	C-Type Lectin (CTL)—mannose binding
AAEL000556-RA	−2.9238	1.4 × 10^−6^	C-Type Lectin (CTL)
AAEL006417-RA	−4.6920	2.9 × 10^−9^	D7 protein
AAEL000726-RA	−3.3941	9.3 × 10^−8^	fibrinogen and fibronectin
AAEL007942-RA	−2.4764	1.1 × 10^−4^	fibrinogen and fibronectin
AAEL011007-RA	−2.9856	1.3 × 10^−4^	fibrinogen and fibronectin
AAEL007064-RA	−2.7633	2.4 × 10^−3^	Gram-Negative Binding Protein (GNBP)
AAEL000576-RA	3.8061	1.3 × 10^−3^	lachesin
AAEL012092-RA	−2.0902	1.7 × 10^−3^	leucine-rich repeat protein
AAEL000243-RA	4.4869	7.5 × 10^−4^	leucine-rich transmembrane protein
AAEL003597-RB	2.3056	2.3 × 10^−3^	leucine-rich transmembrane protein
AAEL007420-RB	−3.1285	1.3 × 10^−4^	Serine Protease Inhibitor (serpin)
AAEL003182-RA	−3.8025	4.7 × 10^−4^	Serine Protease Inhibitor (serpin)
AAEL002704-RB	−4.2338	4.3 × 10^−5^	Serine Protease Inhibitor (serpin)
AAEL010267-RA	4.6569	5.4 × 10^−3^	serine protease
AAEL003508-RB	−2.5065	1.0 × 10^−9^	serine-pyruvate aminotransferase
AAEL001701-RA	−3.9779	6.3 × 10^−3^	serine-type endopeptidase
AAEL008271-RA	6.5329	8.8 × 10^−3^	toll pathway signaling
AAEL007432-RA	6.4799	6.8 × 10^−3^	toll protein
AAEL002510-RB	1.8323	1.2 × 10^−3^	Toll-like receptor
AAEL000224-RA	−3.2320	5.4 × 10^−3^	Toll-like receptor
AAEL007992-RA	−2.0267	2.4 × 10^−3^	trypsin
AAEL005596-RA	−3.6009	1.1 × 10^−5^	trypsin-epsilon
AAEL000793-RA	−3.7994	5.4 × 10^−5^	venom allergen
AAEL006297-RA	−2.6718	1.3 × 10^−3^	venom allergen

**Table 6:** Immunity-related genes significantly regulated in the Key West strain *Aedes aegypti* 3 days Control compared with Control in the Orlando strain (*p*-adj ≤ 0.01, log2-fold change ≥ ± 1.8)**.**

Transcript ID	Log2 FC	*p*-adj	Gene Description
AAEL013063-RA	1.9928	9.8 × 10^−8^	autophagy related gene
AAEL009384-RA	−3.7894	4.5 × 10^−3^	fibrinogen and fibronectin
AAEL009178-RA	−3.8119	2.1 × 10^−4^	Gram-Negative Binding Protein (GNBP)
AAEL009692-RA	1.8392	2.1 × 10^−3^	JAKSTAT pathway signaling signal
AAEL005687-RA	−2.6650	2.9 × 10^−3^	protein serine/threonine kinase
AAEL001914-RA	−2.0191	2.7 × 10^−3^	scavenger receptor
AAEL010267-RA	5.0054	8.3 × 10^−6^	serine protease
AAEL007835-RA	−2.2677	1.1 × 10^−5^	serine/threonine protein kinase
AAEL014188-RA	−2.6299	2.4 × 10^−5^	serine-type endopeptidase
AAEL001701-RA	−3.7945	7.5 × 10^−3^	serine-type endopeptidase
AAEL007992-RA	−3.8714	3.2 × 10^−3^	trypsin
AAEL008080-RA	−2.7126	4.9 × 10^−3^	trypsin-eta
AAEL010486-RA	−2.2017	3.9 × 10^−4^	viral IAP-associated factor

**Table 7:** Multivariate Analysis of Variance (MANOVA) results for strain and time effects of expression of *AaeDEFA, AaeDEFD, AaeDEFa, AaeDNR1, AaeCECH,* and *AaeTEP3* in orally ingested *Ae. aegypti*.

Treatment	Pillai’s Trace	Degrees of Freedom (Numerator, Denominator)	*p*-Value	Standardized Canonical Coefficients					
				*AaeDEFA*	*AaeDEFD*	*AaeDEFa*	*AaeDNR1*	*AaeCECH*	*AaeTEP3*
Strain	0.95	6, 23	<0.0001	2.26	0.53	−1.79	−0.29	0.45	4.48
Time	4.05	36, 168	<0.0001	4.45	2.38	−0.77	−0.39	1.39	−2.34
Strain × Time	3.88	36, 168	<0.0001	5.37	2.95	−0.97	−0.12	−1.57	2.47

**Table 8:** Primers from *Aedes aegypti* used for qPCR reaction.

Gene ID	Accession	Gene Name	Primer Name	Primer Sequence (5’–3’)
AAEL011197	XM_001655125	*Actin*	AaeActin-197-152F	AGGACTCGTACGTCGGTGAC
			AaeActin-197-590R	CGTTCAGTCAGGATCTTC
AAEL003849 ^1^	XM_021856546	*DEFA*	AaeDEF-A-849-208F	CGCCCTTTTGCAAACTCTCT
			AaeDEF-A-849-380R	TTGCAGTAACCTCCCCGATT
AAEL003857	XM_001657239	*DEFD*	AaeDEF-D-857-39F	CACCGGGGCCATTACTAGTG
			AaeDEF-D-857-196R	CGCTCAACAGATCACAGGTG
AAEL003841	XM_001657243	*DEFa*	AaeDEF-A-841-208F	CGCCCTTTTGCAAACTCTCT
			AaeDEF-A-841-380R	TTGCAGTAACCTCCCCGATT
AAEL017211	NW_001809913	*CECH*	AaeCEC-211-131F	CAAGCTGCTATTGGTGGTCG
			AaeCEC-211-312R	CGTTCACGCTTGTCTAAACCA
AAEL008607	XM_021846500	*tep3*	AaeTep3-607-684F	AGTGTCCGTTGAGTCTCCTG
			AaeTep3-607-890R	TCTACCGATCCCTTGCCATC
AAEL000590	XM_001648620	*DNR1*	AaeDNR1-590-550F	AGCATTGCATCGACAGTCAC
			AaeDNR1-590-738R	AGCGGAACTTGCAGTCATTT

^1^ AAEL003849 is the same gene as AAEL027792.

## Supplementary Materials

Supplementary materials can be found at https://www.mdpi.com/1422-0067/20/13/3133/s1. Table S1. Summary of RNA-seq analysis based on the *Aedes aegypti* transcriptomes; Figure S1A–E. GO analyses for RNA-sequencing data.

## Abbreviations

CHIKV	chikungunya virus
CECH	cecropin anti-microbial peptide
DEF	defensin
TEP	thioester-containing proteins

## References

[1] Simo Tchetgna H., Sem Ouilibona R., Nkili-Meyong A.A., Caron M., Labouba I., Selekon B., Njouom R., Leroy E.M., Nakoune E., Berthet N. (2018). Viral Exploration of Negative Acute Febrile Cases Observed during Chikungunya Outbreaks in Gabon. Intervirology.

[2] Lanciotti R.S., Valadere A.M. (2014). Transcontinental movement of Asian genotype chikungunya virus. Emerg. Infect. Dis..

[3] Leparc-Goffart I., Nougairede A., Cassadou S., Prat C., de Lamballerie X. (2014). Chikungunya in the Americas. Lancet.

[4] Tsetsarkin K.A., Chen R., Weaver S.C. (2016). Interspecies transmission and chikungunya virus emergence. Curr. Opin. Virol..

[5] Sudeep A.B., Shil P. (2017). (Bigot) mosquito: An emerging threat to public health. J. Vector Borne Dis..

[6] Ciocchetta S., Prow N.A., Darbro J.M., Frentiu F.D., Savino S., Montarsi F., Capelli G., Aaskov J.G., Devine G.J. (2018). The new European invader *Aedes* (Finlaya) *koreicus*: A potential vector of chikungunya virus. Pathog. Glob. Health.

[7] Powers A.M. (2016). How Chikungunya Virus Virology Affects Its Epidemiology and Transmission: Implications for Influencing Public Health. J. Infect. Dis..

[8] Caglioti C., Lalle E., Castilletti C., Carletti F., Capobianchi M.R., Bordi L. (2013). Chikungunya virus infection: An overview. New Microbiol..

[9] Amdekar S., Parashar D., Alagarasu K. (2017). Chikungunya Virus-Induced Arthritis: Role of Host and Viral Factors in the Pathogenesis. Viral Immunol..

[10] Gasque P., Bandjee M.C., Reyes M.M., Viasus D. (2016). Chikungunya Pathogenesis: From the Clinics to the Bench. J. Infect. Dis..

[11] Hua C., Combe B. (2017). Chikungunya Virus-Associated Disease. Curr. Rheumatol. Rep..

[12] Ausubel F.M. (2005). Are innate immune signaling pathways in plants and animals conserved?. Nat. Immunol..

[13] Flajnik M.F. (2018). A cold-blooded view of adaptive immunity. Nat. Rev. Immunol..

[14] Kumar A., Srivastava P., Sirisena P., Dubey S.K., Kumar R., Shrinet J., Sunil S. (2018). Mosquito Innate Immunity. Insects.

[15] Houk E.J., Hardy J.L., Chiles R.E. (1981). Permeability of the midgut basal lamina in the mosquito, *Culex tarsalis* Coquillett (Insecta, Diptera). Acta Trop..

[16] Passarelli A.L. (2011). Barriers to success: How baculoviruses establish efficient systemic infections. Virology.

[17] Dong S., Behura S.K. (2017). Franz AWE: The midgut transcriptome of *Aedes aegypti* fed with saline or protein meals containing chikungunya virus reveals genes potentially involved in viral midgut escape. BMC Genomics.

[18] Kantor A.M., Dong S., Held N.L., Ishimwe E., Passarelli A.L., Clem R.J., Franz A.W. (2017). Identification and initial characterization of matrix metalloproteinases in the yellow fever mosquito, *Aedes aegypti*. Insect Mol. Biol..

[19] Bartholomay L.C., Cho W.L., Rocheleau T.A., Boyle J.P., Beck E.T., Fuchs J.F., Liss P., Rusch M., Butler K.M., Wu R.C. (2004). Description of the transcriptomes of immune response-activated hemocytes from the mosquito vectors *Aedes aegypti* and *Armigeres subalbatus*. Infect. Immun..

[20] Dimopoulos G., Richman A., Müller H.M., Kafatos F.C. (1997). Molecular immune responses of the mosquito *Anopheles gambiae* to bacteria and malaria parasites. Proc. Natl. Acad. Sci. USA.

[21] Bartholomay L.C., Fuchs J.F., Cheng L.L., Beck E.T., Vizioli J., Lowenberger C., Christensen B.M. (2004). Reassessing the role of defensin in the innate immune response of the mosquito, *Aedes aegypti*. Insect Mol. Biol..

[22] Bartholomay L.C., Michel K. (2018). Mosquito Immunobiology: The Intersection of Vector Health and Vector Competence. Annu. Rev. Entomol..

[23] Dimopoulos G., Seeley D., Wolf A., Kafatos F.C. (1998). Malaria infection of the mosquito *Anopheles gambiae* activates immune-responsive genes during critical transition stages of the parasite life cycle. EMBO J..

[24] Dimopoulos G., Casavant T.L., Chang S., Scheetz T., Roberts C., Donohue M., Schultz J., Benes V., Bork P., Ansorge W. (2000). *Anopheles gambiae* pilot gene discovery project: Identification of mosquito innate immunity genes from expressed sequence tags generated from immune-competent cell lines. Proc. Natl. Acad. Sci. USA.

[25] Osta M.A., Christophides G.K., Vlachou D., Kafatos F.C. (2004). Innate immunity in the malaria vector *Anopheles gambiae*: Comparative and functional genomics. J. Exp. Biol..

[26] Sim S., Jupatanakul N., Ramirez J.L., Kang S., Romero-Vivas C.M., Mohammed H., Dimopoulos G. (2013). Transcriptomic profiling of diverse *Aedes aegypti* strains reveals increased basal-level immune activation in dengue virus-refractory populations and identifies novel virus-vector molecular interactions. PLoS Negl. Trop. Dis..

[27] Etebari K., Hegde S., Saldaña M.A., Widen S.G., Wood T.G., Asgari S., Hughes G.L. (2017). Global Transcriptome Analysis of *Aedes aegypti* Mosquitoes in Response to Zika Virus Infection. mSphere.

[28] Shrinet J., Srivastava P., Sunil S. (2017). Transcriptome analysis of *Aedes aegypti* in response to mono-infections and co-infections of dengue virus-2 and chikungunya virus. Biochem. Biophys. Res. Commun..

[29] Zhao L., Alto B.W., Shin D., Yu F. (2018). The effect of permethrin resistance on *Aedes aegypti* transcriptome following ingestion of Zika virus infected blood. Viruses.

[30] Shin D., Civana A., Acevedo C., Smartt C.T. (2014). Transcriptomics of differential vector competence: West Nile virus infection in two populations of *Culex pipiens quinquefasciatus* linked to ovary development. BMC Genomics.

[31] Shrinet J., Jain S., Jain J., Bhatnagar R.K., Sunil S. (2014). Next generation sequencing reveals regulation of distinct *Aedes* microRNAs during chikungunya virus development. PLoS Negl. Trop. Dis..

[32] Bonizzoni M., Dunn W.A., Campbell C.L., Olson K.E., Marinotti O., James A.A. (2012). Complex modulation of the *Aedes aegypti* transcriptome in response to dengue virus infection. PLoS ONE.

[33] Colpitts T.M., Cox J., Vanlandingham D.L., Feitosa F.M., Cheng G., Kurscheid S., Wang P., Krishnan M.N., Higgs S., Fikrig E. (2011). Alterations in the *Aedes aegypti* transcriptome during infection with West Nile, dengue and yellow fever viruses. PLoS Pathog..

[34] Wang H., Smagghe G., Meeus I. (2017). The role of a single gene encoding the Single von Willebrand factor C-domain protein (SVC) in bumblebee immunity extends beyond antiviral defense. Insect Biochem. Mol. Biol..

[35] Zhao L., Alto B.W., Smartt C.T., Shin D. (2017). Transcription Profiling for Defensins of *Aedes aegypti* (Diptera: Culicidae) During Development and in Response to Infection with Chikungunya and Zika Viruses. J. Med. Entomol..

[36] Antonova Y., Alvarez K.S., Kim Y.J., Kokoza V., Raikhel A.S. (2009). The role of NF-kappaB factor REL2 in the *Aedes aegypti* immune response. Insect Biochem. Mol. Biol..

[37] Kokoza V., Ahmed A., Woon Shin S., Okafor N., Zou Z., Raikhel A.S. (2010). Blocking of *Plasmodium* transmission by cooperative action of Cecropin A and Defensin A in transgenic *Aedes aegypti* mosquitoes. Proc. Natl. Acad. Sci. USA.

[38] Angleró-Rodríguez Y.I., MacLeod H.J., Kang S., Carlson J.S., Jupatanakul N., Dimopoulos G. (2017). Molecular Responses to Zika Virus: Modulation of Infection by the Toll and Jak/Stat Immune Pathways and Virus Host Factors. Front. Microbiol..

[39] Zhao L., Alto B.W., Shin D. (2019). Transcriptional profile of *Aedes aegypti* Leucine-Rich Repeat Proteins in response to Zika and Chikungunya viruses. Int. J. Mol. Sci..

[40] Aloor J.J., Azzam K.M., Guardiola J.J., Gowdy K.M., Madenspacher J.H., Gabor K.A., Mueller G.A., Lin W.C., Lowe J.M., Gruzdev A. (2019). Leucine-Rich Repeats and Calponin Homology containing 4 regulates the innate immune response. J. Biol. Chem..

[41] Reyes Ruiz V.M., Sousa G.L., Sneed S.D., Farrant K.V., Christophides G.K., Povelones M. (2019). Stimulation of a protease targeting the LRIM1/APL1C complex reveals specificity in complement-like pathway activation in *Anopheles gambiae*. PLoS ONE.

[42] Souza-Neto J.A., Sim S., Dimopoulos G. (2009). An evolutionary conserved function of the JAK-STAT pathway in anti-dengue defense. Proc. Natl. Acad. Sci. USA.

[43] Xi Z., Ramirez J.L., Dimopoulos G. (2008). The *Aedes aegypti* toll pathway controls dengue virus infection. PLoS Pathog..

[44] Yan Y., Hillyer J.F. (2019). Complement-like proteins TEP1, TEP3 and TEP4 are positive regulators of periostial hemocyte aggregation in the mosquito *Anopheles gambiae*. Insect Biochem. Mol. Biol..

[45] Soares T.S., Rodriguez Gonzalez B.L., Torquato R.J.S., Lemos F.J.A., Costa-da-Silva A.L., Capurro Guimarães M.L., Tanaka A.S. (2018). Functional characterization of a serine protease inhibitor modulated in the infection of the *Aedes aegypti* with dengue virus. Biochimie.

[46] Gaburro J., Paradkar P.N., Klein M., Bhatti A., Nahavandi S., Duchemin J.B. (2018). Dengue virus infection changes *Aedes aegypti* oviposition olfactory preferences. Sci. Rep..

[47] Gaburro J., Bhatti A., Sundaramoorthy V., Dearnley M., Green D., Nahavandi S., Paradkar P.N., Duchemin J.B. (2018). Zika virus-induced hyper excitation precedes death of mouse primary neuron. Virol. J..

[48] Sim S., Ramirez J.L., Dimopoulos G. (2012). Dengue virus infection of the *Aedes aegypti* salivary gland and chemosensory apparatus induces genes that modulate infection and blood-feeding behavior. PLoS Pathog..

[49] Bonizzoni M., Dunn W.A., Campbell C.L., Olson K.E., Marinotti O., James A.A. (2012). Strain Variation in the Transcriptome of the Dengue Fever Vector, *Aedes aegypti*. G3 Genes Genomes Genet. (Bethesda).

[50] Parola P., de Lamballerie X., Jourdan J., Rovery C., Vaillant V., Minodier P., Brouqui P., Flahault A., Raoult D., Charrel R.N. (2006). Novel chikungunya virus variant in travelers returning from Indian Ocean islands. Emerg. Infect. Dis..

[51] Kim C.H., Muturi E.J. (2013). Effect of larval density and Sindbis virus infection on immune responses in *Aedes aegypti*. J. Insect Physiol..

[52] Muturi E.J., Blackshear M., Montgomery A. (2012). Temperature and density-dependent effects of larval environment on *Aedes aegypti* competence for an alphavirus. J. Vector Ecol..

[53] Pan X., Zhou G., Wu J., Bian G., Lu P., Raikhel A.S., Xi Z. (2012). *Wolbachia* induces reactive oxygen species (ROS)-dependent activation of the Toll pathway to control dengue virus in the mosquito *Aedes aegypti*. Proc. Natl. Acad. Sci. USA.

[54] Yao J.Q., Yu F. (2011). DEB: A web interface for RNA-seq digital gene expression analysis. Bioinformation.

[55] Zhao L., Alto B.W., Duguma D. (2017). Transcriptional Profile for Detoxification Enzymes AeaGGT1 and AaeGGT2 from *Aedes aegypti* (Diptera: Culicidae) in Response to Larvicides. J. Med. Entomol..

[56] Zhao L., Pridgeon J.W., Becnel J.J., Clark G.G., Linthicum K.J. (2008). Cytochrome c gene and protein expression: Developmental regulation, environmental response, and pesticide sensitivity in *Aedes aegypti*. J. Med. Entomol..

[57] Portereiko M.F., Sandaklie-Nikolova L., Lloyd A., Dever C.A., Otsuga D., Drews G.N. (2006). NUCLEAR FUSION DEFECTIVE1 encodes the Arabidopsis RPL21M protein and is required for karyogamy during female gametophyte development and fertilization. Plant Physiol..

[58] Livak K.J., Schmittgen T.D. (2001). Analysis of relative gene expression data using real-time quantitative PCR and the 2^−ΔΔ*C*T^ Method. Methods.

[59] Portereiko M.F., Lloyd A., Steffen J.G., Punwani J.A., Otsuga D., Drews G.N. (2006). AGL80 is required for central cell and endosperm development in *Arabidopsis*. Plant Cell.

