# Supplementary material for: Transcriptomic Analysis of Aedes aegypti Innate Immune System in Response to Ingestion of Chikungunya Virus
Source: Int J Mol Sci. 2019 Jun 27;20(13):3133. doi: 10.3390/ijms20133133 (PMC6651163; doi:10.3390/ijms20133133)
Supplement: Supplementary file 1 [file ijms-20-03133-s001.pdf]

Supplementary Materials

Transcriptomic Analysis of *Aedes aegypti* Innate Immune System in Response to Ingestion of Chikungunya Virus

Liming Zhao [1,*], Barry W. Alto [1], Yongxing Jiang [2], Fahong Yu [3], and Yanping Zhang [3]

[1] Florida Medical Entomology Laboratory, University of Florida, 200 9th Street South East, Vero Beach, FL 32962, USA; bwalto@ufl.edu
[2] Mosquito Control Services, City of Gainesville, 405 NW 39th Avenue Gainesville, FL 32609, USA; JiangY1@cityofgainesville.org
[3] Interdisciplinary Center for Biotechnology Research, University of Florida, 2033 Mowry Road, Gainesville, FL 32611, USA; fyu@ufl.edu (F.Y.); yanp@ufl.edu (Y.Z.)
* Correspondence: lmzhao@ufl.edu; Tel.: +01-772-778-7200

Received: 21 May 2019; Accepted: 23 June 2019; Published: 26 June 2019

Figure S1 (A–E). GO analyses for RNA-seq data. Overview of the functional categories of differentially expressed (DE) transcripts in response to CHIKV infection. DE genes were determined based on statistical analysis by DESeq package. The total number of DE genes for each comparison is shown in parentheses in each figure. Gene ontology analysis of DE genes was performed based on the database of AmiGO 2. Up, upregulated DE genes; Down, downregulated DE genes. A. 3 hours post injection KW-CHIKV compared with KW-Control; B. 3-day post infection, KW-CHIKV compared with KW-Control; C. 3-day post injection, KW-Control compared with OR-Control; D. 3-day post infection, KW-CHIKV compared with OR-CHIKV; E. 3-day post infection, E1. OR-CHIKV compared with OR-Control; E2. OR-CHIKV compared with OR-Control 339 gene down regulated with *padj* ≤ 0.01 only three gens were detected in the GO, which not shown in the figure.

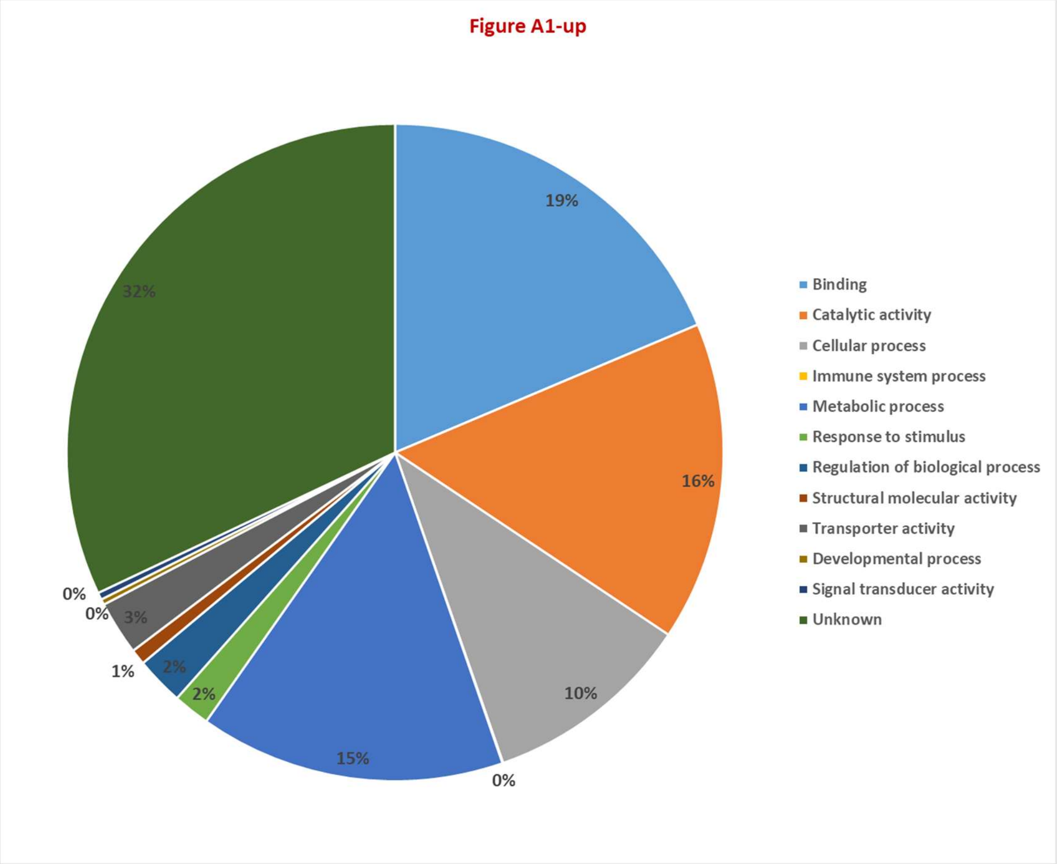

Functional categories		Gene No.	Percentage
GO:0005488	Binding	242	18.64%
GO:0003824	Catalytic activity	204	15.71%
GO:0009987	Cellular process	134	10.31%
GO:0002376	Immune system process	0	0.00%
GO:0008152	Metabolic process	196	15.05%
GO:0050896	Response to stimulus	24	1.82%
GO:0050789	Regulation of biological process	31	2.37%
GO:0005198	Structural molecular activity	10	0.77%
GO:0005215	Transporter activity	34	2.65%
GO:0032502	Developmental process	4	0.29%
GO:0004871	Signal transducer activity	5	0.34%
		883	
	Unknown	416	32.02%
Figure S1A1	Total	1299	

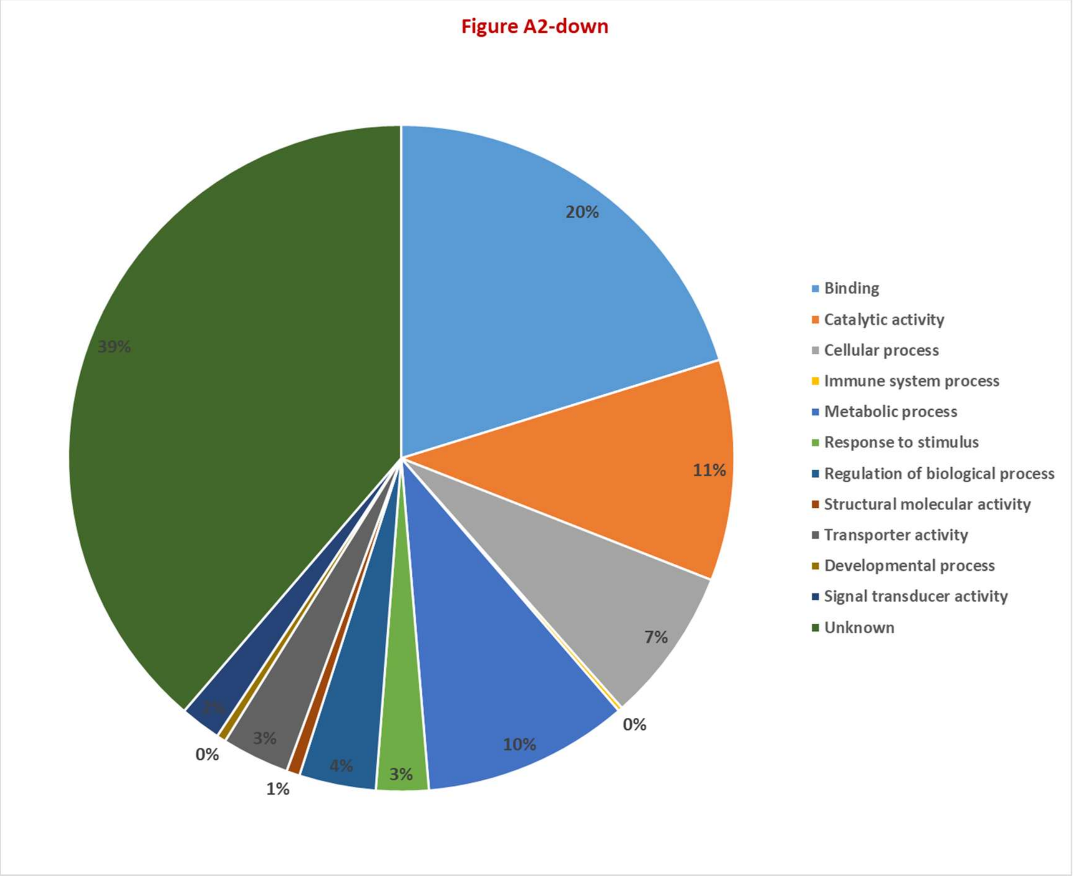

Functional categories		Gene No.	Percentage
GO:0005488	Binding	246	20.23%
GO:0003824	Catalytic activity	130	10.72%
GO:0009987	Cellular process	92	7.53%
GO:0002376	Immune system process	2	0.20%
GO:0008152	Metabolic process	122	9.99%
GO:0050896	Response to stimulus	31	2.55%
GO:0050789	Regulation of biological process	45	3.72%
GO:0005198	Structural molecular activity	8	0.65%
GO:0005215	Transporter activity	40	3.28%
GO:0032502	Developmental process	5	0.45%
GO:0004871	Signal transducer activity	24	1.98%
		746	
	Unknown	471	38.70%
Figure S1A2	Total	1217	

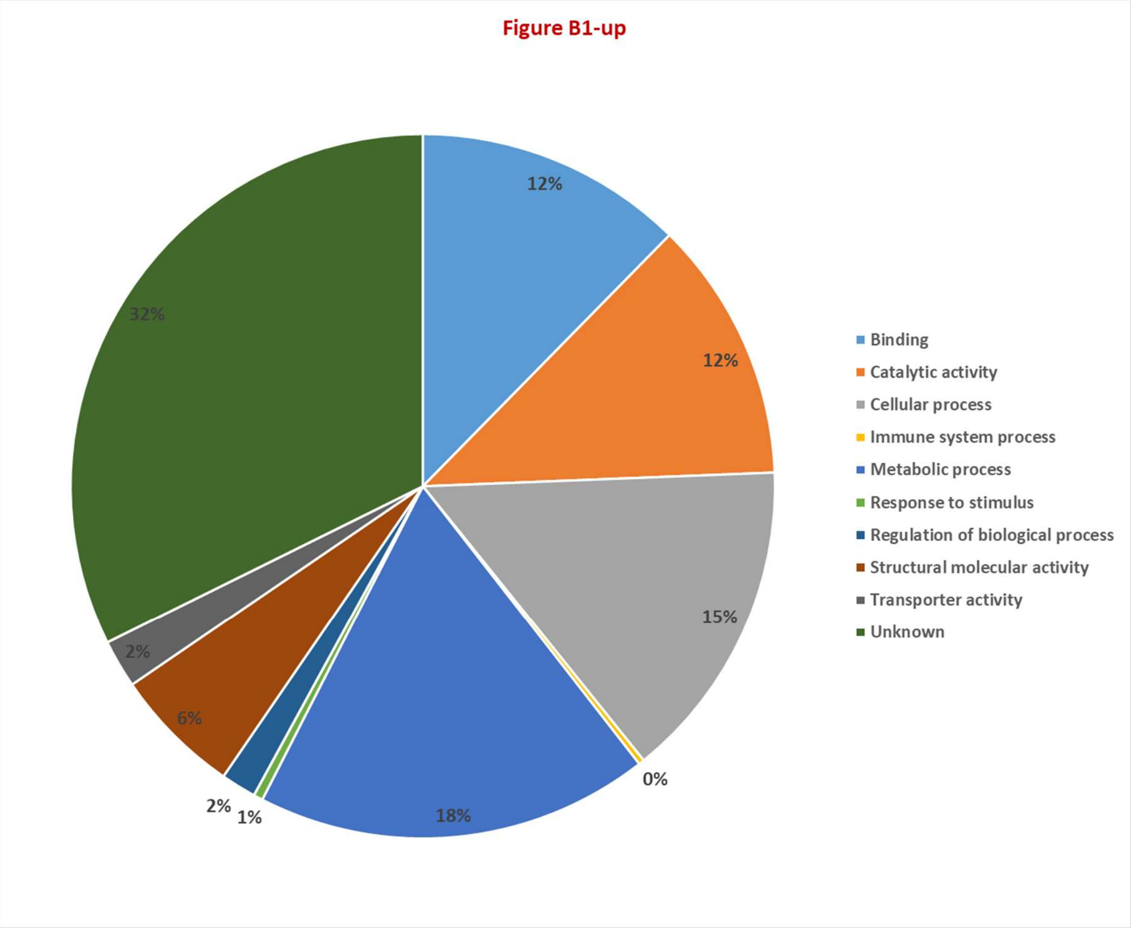

Functional categories		Gene No.	Percentage
GO:0005488	Binding	65	13.11%
GO:0003824	Catalytic activity	63	12.75%
GO:0009987	Cellular process	78	15.73%
GO:0002376	Immune system process	1	2.71%
GO:0008152	Metabolic process	95	19.16%
GO:0050896	Response to stimulus	2	0.45%
GO:0050789	Regulation of biological process	9	1.72%
GO:0005198	Structural molecular activity	31	6.24%
GO:0005215	Transporter activity	12	2.35%
GO:0032502	Developmental process	0	0.00%
GO:0004871	Signal transducer activity	0	0.00%
		356	
	Unknown	170	34.27%
Figure S1B1	Total	496	

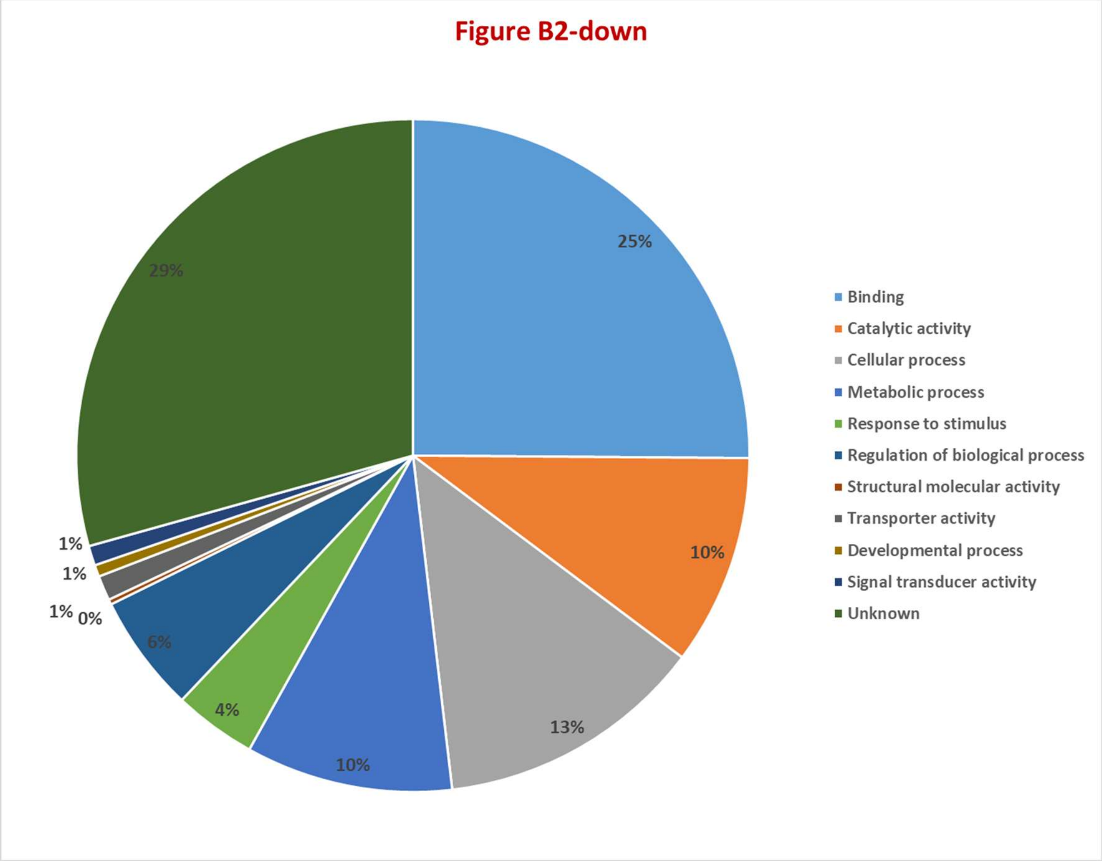

Functional categories		Gene No.	Percentage
GO:0005488	Binding	308	25.13%
GO:0003824	Catalytic activity	124	10.12%
GO:0009987	Cellular process	158	12.90%
GO:0002376	Immune system process	0	0.00%
GO:0008152	Metabolic process	122	9.94%
GO:0050896	Response to stimulus	49	3.97%
GO:0050789	Regulation of biological process	69	5.66%
GO:0005198	Structural molecular activity	3	0.25%
GO:0005215	Transporter activity	15	1.19%
GO:0032502	Developmental process	7	0.56%
GO:0004871	Signal transducer activity	12	0.95%
		867	
	Unknown	360	29.34%
Figure S1B2	Total	1227	

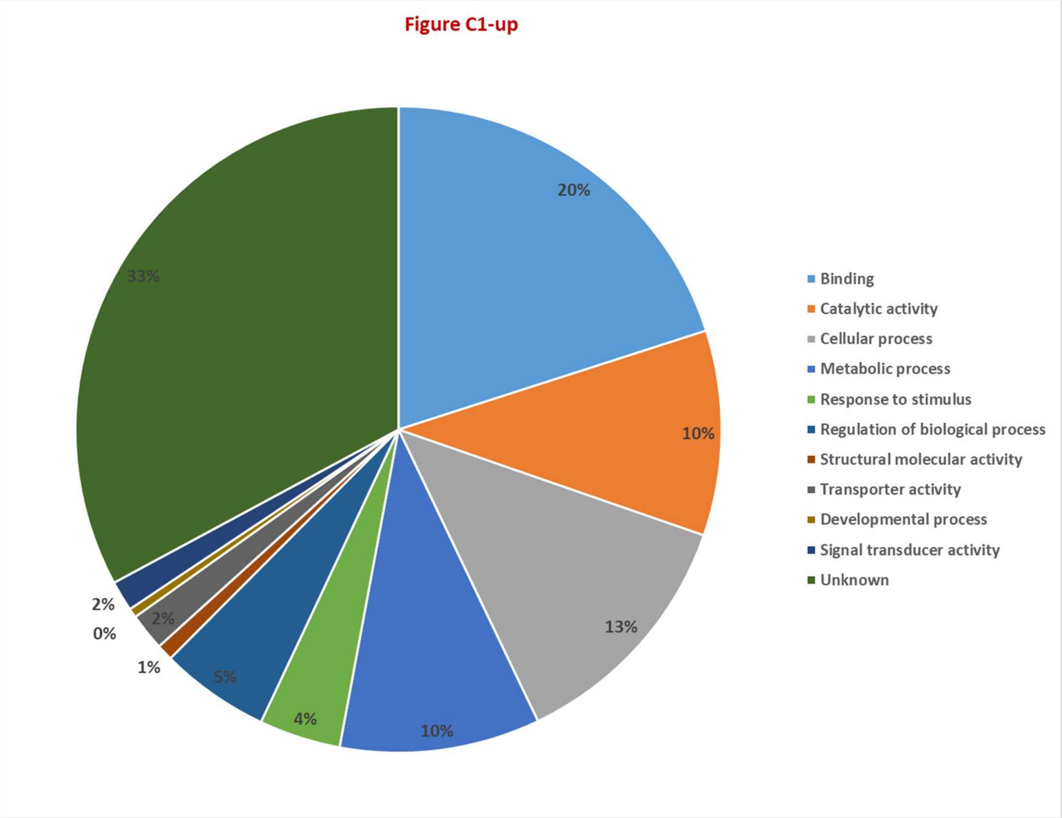

Functional categories		Gene No.	Percentage
GO:0005488	Binding	70	20.05%
GO:0003824	Catalytic activity	36	10.26%
GO:0009987	Cellular process	44	12.59%
GO:0002376	Immune system process	0	0.00%
GO:0008152	Metabolic process	35	10.03%
GO:0050896	Response to stimulus	14	4.08%
GO:0050789	Regulation of biological process	19	5.48%
GO:0005198	Structural molecular activity	3	0.82%
GO:0005215	Transporter activity	7	1.87%
GO:0032502	Developmental process	2	0.47%
GO:0004871	Signal transducer activity	5	1.52%
		235	
	Unknown	115	32.85%
Figure S1C1	Total	350	

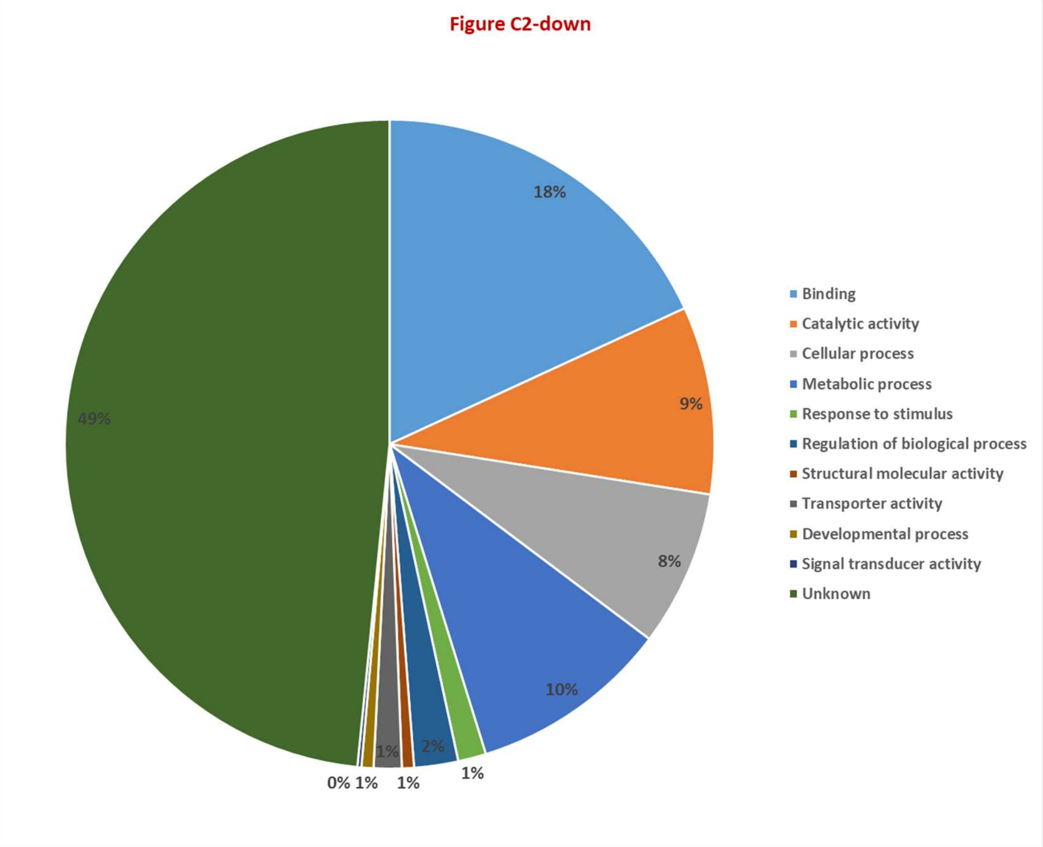

Functional categories		Gene No.	Percentage
GO:0005488	Binding	46	18.13%
GO:0003824	Catalytic activity	24	9.36%
GO:0009987	Cellular process	20	7.77%
GO:0002376	Immune system process	0	0.00%
GO:0008152	Metabolic process	25	9.96%
GO:0050896	Response to stimulus	4	1.39%
GO:0050789	Regulation of biological process	6	2.19%
GO:0005198	Structural molecular activity	2	0.60%
GO:0005215	Transporter activity	4	1.39%
GO:0032502	Developmental process	2	0.60%
GO:0004871	Signal transducer activity	1	0.20%
		130	
	Unknown	122	48.41%
**Figure S1C2**	Total	252	

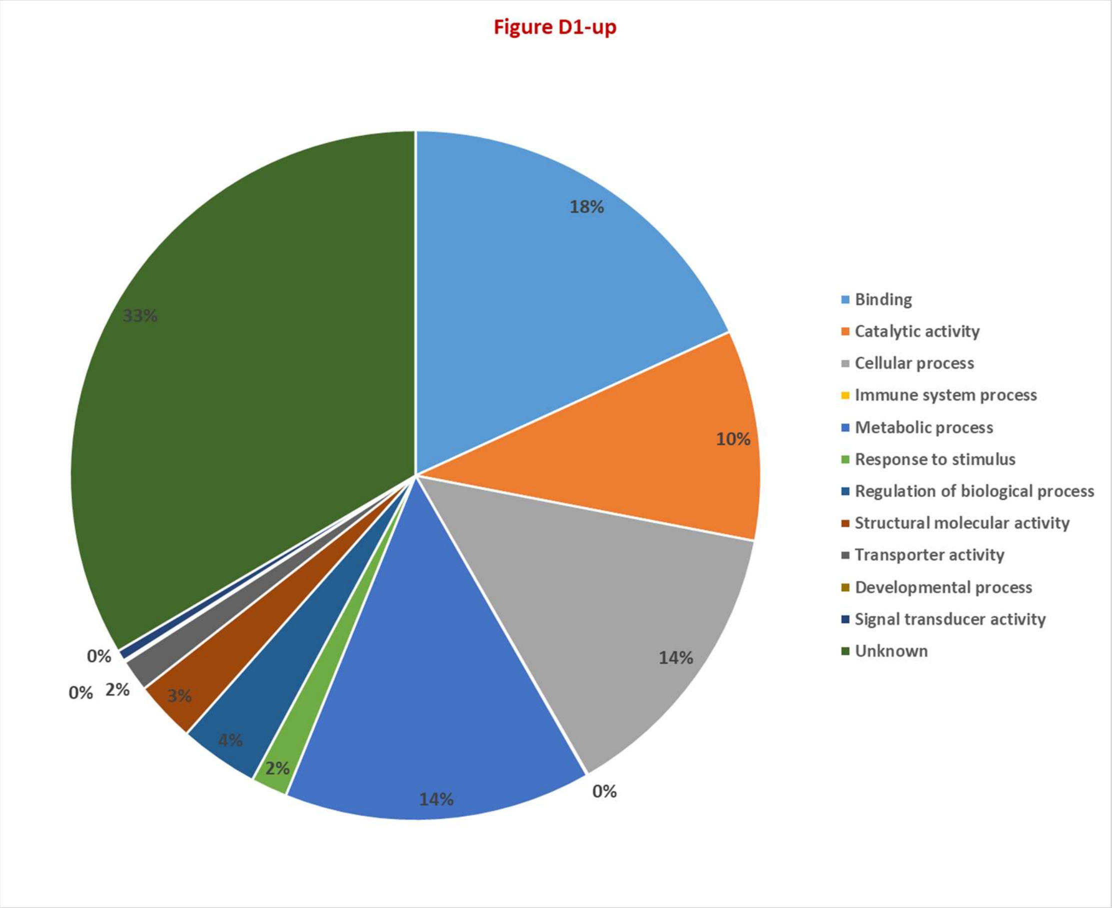

Functional categories		Gene No.	Percentage
GO:0005488	Binding	156	18.16%
GO:0003824	Catalytic activity	85	9.88%
GO:0009987	Cellular process	117	13.64%
GO:0002376	Immune system process	0	0.00%
GO:0008152	Metabolic process	124	14.40%
GO:0050896	Response to stimulus	15	1.71%
GO:0050789	Regulation of biological process	32	3.71%
GO:0005198	Structural molecular activity	25	2.86%
GO:0005215	Transporter activity	13	1.50%
GO:0032502	Developmental process	1	0.10%
GO:0004871	Signal transducer activity	4	0.50%
		572	
	Unknown	288	48.41%
Figure S1D1	Total	860	

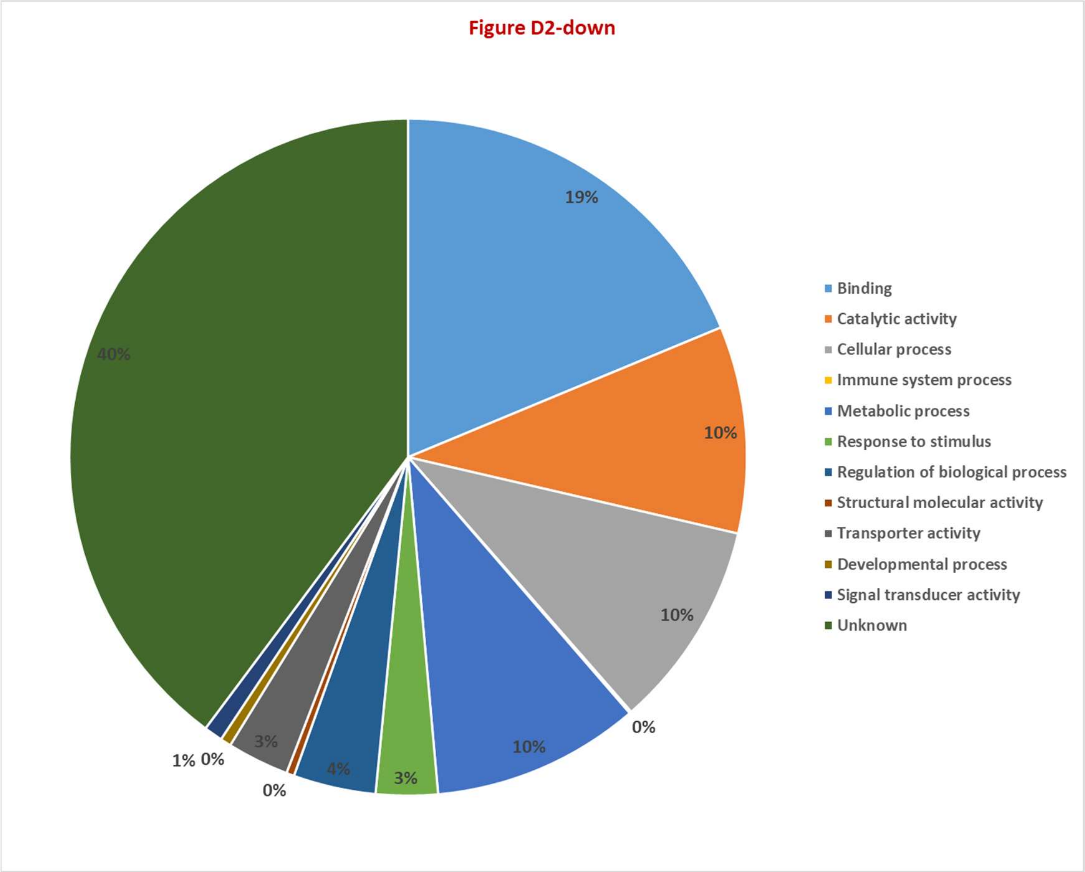

Functional categories		Gene No.	Percentage
GO:0005488	Binding	183	18.74%
GO:0003824	Catalytic activity	96	9.88%
GO:0009987	Cellular process	97	9.93%
GO:0002376	Immune system process	1	0.09%
GO:0008152	Metabolic process	97	9.93%
GO:0050896	Response to stimulus	29	2.95%
GO:0050789	Regulation of biological process	38	3.94%
GO:0005198	Structural molecular activity	4	0.37%
GO:0005215	Transporter activity	29	2.95%
GO:0032502	Developmental process	5	0.52%
GO:0004871	Signal transducer activity	9	0.89%
		587	
	Unknown	388	39.79%
Figure S1D2	Total	975	

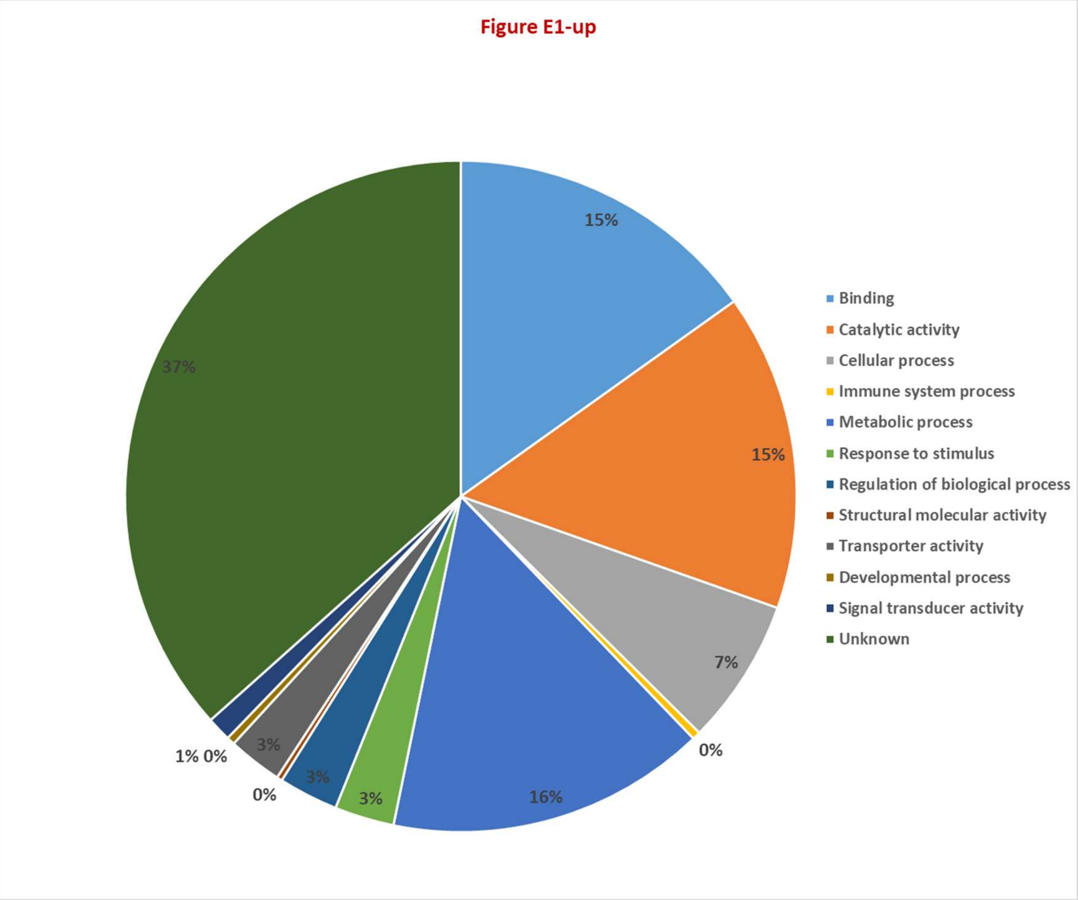

Functional categories		Gene No.	Percentage
GO:0005488	Binding	51	15.13%
GO:0003824	Catalytic activity	51	15.26%
GO:0009987	Cellular process	24	7.04%
GO:0002376	Immune system process	1	0.39%
GO:0008152	Metabolic process	52	15.39%
GO:0050896	Response to stimulus	10	2.87%
GO:0050789	Regulation of biological process	10	2.87%
GO:0005198	Structural molecular activity	1	0.26%
GO:0005215	Transporter activity	9	2.61%
GO:0032502	Developmental process	1	0.39%
GO:0004871	Signal transducer activity	4	1.17%
		213	
	Unknown	123	36.61%
**Figure S1E1**	Total	336	

Table S1. Summary of RNA-seq analysis based on the *Aedes aegypti* transcriptomes.

Sample ID	Raw reads R1	Raw reads R2	Cleaned reads R1	Cleaned reads R1	Mapped reads (R1+R2)	% of mapped reads	Mapped transcripts
A3 [1]	15,249,374	15,249,374	15,246,445	15,197,063	16,456,732	54.06	13,340
B3 [1]	14,551,995	14,551,995	14,547,933	14,507,126	17,859,022	61.47	13,471
C3 [1]	15,681,057	15,681,057	15,679,156	15,660,510	19,742,347	62.99	13,985
E3 [2]	18,294,687	18,294,687	18,292,355	18,208,093	22,039,021	60.38	14,386
F3 [2]	12,497,295	12,497,295	12,495,450	12,455,741	15,396,499	61.71	13,952
G3 [2]	17,566,008	17,566,008	17,564,008	17,531,085	21,793,543	62.10	14,379
H3 [3]	36,035,055	36,035,055	36,024,025	36,011,050	25,633,634	35.58	14,287
I3 [3]	36,757,692	36,757,692	36,739,213	36,724,336	35,906,241	48.88	14,552
J3 [3]	32,925,593	32,925,593	32,914,213	32,901,086	36,273,185	55.11	14,065
K3 [4]	30,895,140	30,895,140	30,887,834	30,883,906	15,714,415	25.44	13,699
L3 [4]	29,453,796	29,453,796	29,439,544	29,428,165	30,662,321	52.09	13,957
M3 [4]	32,527,728	32,527,728	32,520,115	32,506,621	32,313,251	49.69	14,226
H0 [5]	31,187,794	31,187,794	31,185,876	31,171,790	9,822,781	15.75	13,506
I0 [5]	30,743,396	30,743,396	30,738,833	30,725,609	32,979,060	53.66	14,376
J0 [5]	33,318,113	33,318,113	33,310,791	33,296,664	37,160,333	55.79	14,026
K0 [6]	37,018,624	37,018,624	37,009,861	36,994,319	43,186,699	58.36	14,879
L0 [6]	33,265,010	33,265,010	33,260,119	33,247,298	34,456,987	51.81	14,755
M0 [6]	34,732,453	34,732,453	34,727,178	34,712,231	22,215,700	31.99	14,423

[1] A3, B3, and C3 are *Aedes aegypti* Orlando strain control three-days post ingection blood;

[2] E3, F3, and G3 are *Ae. aegypti* Orlando strain chikungunya virus three-days post ingection;

[3] H3, I3, and J3 are *Ae. aegypti* Key West strain control three-days post ingection blood;

[4] K3, L3, and M3 are *Ae. aegypti* Key West strain chikungunya virus three-days post ingection;

[5] H0, I0 and J0 are *Ae. aegypti* Key West strain control three-hours post ingection blood;

[6] K0, L0 and M0 are *Ae. aegypti* Key West strain chikungunya virus three-hours post ingection.
